# Predicting subjective well-being in a high-risk sample of Russian mental health app users

**DOI:** 10.1140/epjds/s13688-022-00333-x

**Published:** 2022-04-04

**Authors:** Polina Panicheva, Larisa Mararitsa, Semen Sorokin, Olessia Koltsova, Paolo Rosso

**Affiliations:** 1 Laboratory for Social and Cognitive Informatics, HSE University, Saint Petersburg, Russia; 2 Humanteq, Moscow, Russia; 3 Pattern Recognition and Human Language Technology Research Center, Universitat Politècnica de València, Valencia, Spain

**Keywords:** Digital traces, Subjective well-being, Mental health prediction

## Abstract

Despite recent achievements in predicting personality traits and some other human psychological features with digital traces, prediction of subjective well-being (SWB) appears to be a relatively new task with few solutions. COVID-19 pandemic has added both a stronger need for rapid SWB screening and new opportunities for it, with online mental health applications gaining popularity and accumulating large and diverse user data. Nevertheless, the few existing works so far have aimed at predicting SWB, and have done so only in terms of Diener’s Satisfaction with Life Scale. None of them analyzes the scale developed by the World Health Organization, known as WHO-5 – a widely accepted tool for screening mental well-being and, specifically, for depression risk detection. Moreover, existing research is limited to English-speaking populations, and tend to use text, network and app usage types of data separately. In the current work, we cover these gaps by predicting both mentioned SWB scales on a sample of Russian mental health app users who represent a population with high risk of mental health problems. In doing so, we employ a unique combination of phone application usage data with private messaging and networking digital traces from VKontakte, the most popular social media platform in Russia. As a result, we predict Diener’s SWB scale with the state-of-the-art quality, introduce the first predictive models for WHO-5, with similar quality, and reach high accuracy in the prediction of clinically meaningful classes of the latter scale. Moreover, our feature analysis sheds light on the interrelated nature of the two studied scales: they are both characterized by negative sentiment expressed in text messages and by phone application usage in the morning hours, confirming some previous findings on subjective well-being manifestations. At the same time, SWB measured by Diener’s scale is reflected mostly in lexical features referring to social and affective interactions, while mental well-being is characterized by objective features that reflect physiological functioning, circadian rhythms and somatic conditions, thus saliently demonstrating the underlying theoretical differences between the two scales.

## Introduction

In recent years, evaluation, analysis and improvement of subjective well-being (SWB) has gained a growing attention of both researchers and practitioners [1, 2]. Attention to SWB has naturally been coupled with the increasing research interest in depression – the leading cause of disability and subjective well-being loss worldwide [3, 4]. The COVID-19 pandemic, resulting in the shift to hybrid work and the decline in face-to-face communication has put many individuals at additional mental health risks [5, 6]. Some of the most widely available instruments to mitigate such risks are online and mobile services that offer quick screening tests of subjective well-being and mental health states and automatically generate respective recommendations. More than 240 mental health apps are available in the App Store today, some of which are extensively using machine learning for classifying and scoring their users in terms of their psychological or mental conditions [7–9]. Such apps attract consumers concerned with their psychological states, while these concerns are usually associated with higher risks for users’ SWB or mental health. As these individuals agree to donate parts of their digital traces, psychological apps become natural hubs accumulating data on individuals at risk. Such data, if available, provide ample opportunities for the development of open source algorithms for early automatic detection of threats to well-being in high-risk populations with their digital traces.

Subjective well-being is most commonly defined in accordance with Diener’s approach [10] as a person’s satisfaction with their life (which constitutes SWB’s cognitive component) and the prevalence of positive emotions over negative ones (affective balance, which constitutes SWB’s affective component). To date, about 100 assessment tools measuring about 200 facets of well-being have been proposed, thus complicating the selection of relevant metrics [1]. The two most widely used SWB measurement tools are Diener’s Satisfaction with Life Scale (SWLS) [10] and the scale introduced by the World Health Organization in 1998, known as the WHO-5 index [11]. The former aims to capture generalized long-term subjective well-being, while the original goal of the latter was to screen and rate depression. Later, Bech, one of the WHO-5 developers, also showed that this scale is equally good at detecting high degrees of psychological well-being, which he proposed to consider a component of mental health, along with the absence of depression symptoms [12].

Both SWLS and WHO-5 are short unidimensional 5-item scales with proven validity and reliability (*α* coefficients 0.79–0.89 for the former and 0.82–0.95 for the latter) [13–15]. Both have become common for well-being screening in a wide range of populations and among different nationalities [15–18]. The wide use and the proven quality of these metrics defines their choice for our research in automatic SWB prediction; however, some more details on their distinctive features should be added.

SWLS, apart from being centered on pleasure and satisfaction, is also meant to be time- and dimension-independent. The first feature means that it is not tied to a specific time interval and measures satisfaction with our past, present and future. The second feature refers to the generalized character of such satisfaction, not being tied to any particular dimension of human life, such as health, relationships or finance. The choice of the dimensions to be taken into account and the weight assigned to them is left with the subject and is expected to be based on a blend of objective reality and the subject’s subjective experience of it. It is assumed that a person is able to adequately assess her well-being and has all the necessary and unbiased information for that [10].

SWLS is widely used by psychologists, public health professionals, and economists. According to the World Happiness Report, SWLS provides a more informative measure for international comparisons of well-being than some measures capturing affective component only [19]. Importantly, SWLS is stable under unchanging conditions, but is sensitive to changes in life circumstances: thus,its growth is associated with higher likelihood of marriage and childbirth and with lower likelihood of job loss and relocating [20]. It is also predictive of physical and physiological outcomes, as judged from a 4-year follow-up period in the same study. It is these meaningful changes that have been found responsible for the drop of SWLS test-retest reliability from 0.84 in the window of a few weeks to 0.54 in the 4-year window [21]. These changes are clearly distinct from the short-term random mood fluctuations responsible for explaining 16% of variance in the short run. It thus can said that SWLS captures a stable and a transient components both of which are present in human well-being.

In contrast to SWLS, WHO-5 index aims at a brief assessment of emotional well-being over a 14-day period (thus containing no cognitive component and being highly time-sensitive). Its items represent positive affect whose absence corresponds to the depression symptoms (negative affect). This is an important advantage of WHO-5 as the subjects are not forced to confess of the presence of any unpleasant and potentially hard-to-admit negative emotions or states. As mentioned above, WHO-5 has been proven effective for the detection of both depression risk [22, 23] and the high levels of well-being[12]. Being a short, sensitive, specific and non-invasive tool, it gains over more detailed, but heavier methods for preliminary depression and suicide risk assessment in settings without psychological/psychiatric expertise. WHO-5 has shown high clinimetric validity and the ability to accurately predict a wide range of mental health conditions, including depression; moreover, it has been recommended as an outcome measure balancing the wanted and unwanted effects of treatments [24]. That is why WHO-5 has been adopted in many research fields such as suicidology, geriatrics, youth and alcohol abuse studies, personality disorder research, and occupational psychology [15, 24].

Thus, WHO-5 and SWLS, being psychometrically sound screening tools with known outcomes, also measure complementary aspects of subjective well-being. Although measures of emotional affect and reported life satisfaction often correlate, substantial divergences have been found. For instance, almost half of the people who rated themselves as ‘completely satisfied’ also reported significant symptoms of anxiety and distress [17]. Therefore, quality of life in the current coronavirus crisis is usually measured with both scales [5, 6, 25–27]: while WHO-5 helps to assess influence of different practices on SWB and the persistence of diminished well-being beyond and during COVID-19, SWLS shows how people feel and how their life perspective changes due to the pandemic. This complementarity indicates the importance of comparative research in prediction of both metrics.

This task is novel for SWB prediction with digital traces: despite the advances in detection of specific mental health problems and the attempts to predict some SWB metrics, no research so far has been dedicated to predicting WHO-5 and its comparison with SWLS in terms of digital behavior traces; moreover, most research is limited to English-speaking populations. Best models predicting SWLS with digital traces from social media, search engine and smartphone activity data demonstrate performance below 0.4 in terms of Pearson correlation – a well-known threshold for correlation between psychological characteristics and objective behavior [28, 29] (see also [30, 31] for an overview). None of the models combines language, social media and smartphone usage data.

The goal of this study is to predict individual WHO-5 and SWLS levels with a new combination of digital traces in a high-risk Russian-speaking population, to find out which features are the most predictive and what the overall predictive power of our models is. A high-risk population is defined as a population with a higher probability of having problematic levels of SWB, as compared to more general populations. We thus address a completely novel task of comparative prediction of two different aspects of subjective well-being, which should have different objective indicators and suggest different actions to be taken by the user. Additionally, we find out that depression risk in Russian-speaking population can be detected by the level of WHO-5 below a certain threshold as successfully as in the populations for which WHO-5 was tested earlier, and this allows us to predict the threshold as well. To do so, we make use of a sample of 372 psychological application users who have explicitly consented to share their private messages, social media data and mobile device usage traces. We use extensive feature engineering combined with regression and classification modeling, the first type of models being aimed at SWB score prediction, and the second – and depression risk identification based on theoretically justified thresholds. We also check our regression models against newest neural network approaches that, however, do not show sufficient quality at the dataset of our size.

The rest of the paper is structured as follows. In the next section we review the existing literature in prediction of SWB and related psychological and mental health phenomena with digital traces. Next, we describe our dataset, our numerous features and the approach to their engineering, as well as the models used. In the Results section we report our best models’ performance and the most useful features. In the Discussion section we interpret our results and indicate the most important limitations. We conclude with the perspectives for future research.

### Subjective well-being prediction

Prediction of internal psychological and mental states from objective behavior pattern is a highly difficult task [29, 32]. Additionally, clinically diagnosed mental disorders (such as depression) and mental disorder risks assessed through threshold scores of screening tests (such as WHO-5) are different categories for prediction. While the former may be partially manifest, the latter, along with psychological traits and conditions, are latent constructs. This means that psychological theory does not expect them to fully correlate with any observable patterns since the former are not thought of as reducible to the latter in principle. This may be one of the reasons why such correlation is seldom high, although this is a subject for further research. As both high SWB and the absence of mental disorder symptoms have been shown to be components of mental health [12, 33], prediction of both SWB and mental disorder (or its risk) constitutes two related tasks. However, due to the different nature of SWB and mental disorder as concepts, the former is usually evaluated with continuous predictive models, while the detection of the latter is most often formulated as a classification task.

#### Detection of mental disorders

A vast amount of studies predict specific mental health conditions with digital traces, mostly with the data from social media, such as Facebook and Twitter. The most widely analyzed conditions of such studies are depression and Post Traumatic Stress Disorder [34–38]. Other conditions include Bipolar Disorder, Anxiety and Social Anxiety Disorder, eating disorders, self-harm and suicide attempt [39–42]. Linguistic features used typically include word n-grams, sentiment, specific lexica (e.g., Linguistic Inquiry & Word Count dictionary, LIWC) and topic modelling, with other features related to social networks, emotions, cognitive styles, user activity and demographics [34–39, 42]. Model evaluation metrics include Area Under the Curve (AUC), Precision, Accuracy of classification, and Correlation for continuous measurements. The results for binary mental health problem identification are high, reaching an AUC of 0.7–0.89, Precision up to 0.85, and Accuracy of 0.69–0.72 [30].

Ground truth information in such studies is obtained from different sources, leading to different quality. Most studies use either self-reported survey data [34, 37] or self-declared mental illness [36, 39]. The latter is prone to errors and bias induced by specific data collection methods.

In a recent study Eichstaedt et al. [38] effectively predict depression of Facebook users against medical records information. The authors use a 6-month history of Facebook statuses posted by 683 hospital patients, of whom 114 were diagnosed with depression (rate similar to the general population), and classify depression VS other medical diagnoses with an AUC = 0.72. Features of Facebook statuses include words and word bigrams, temporal characteristics of posting activity, metainformation on post length and frequency, topics and dictionary categories, with interpersonal, emotional and cognitive categories being among the best predictors.

The effects of smartphone usage on mental disorders, until very recently, have been mostly studied with self-reported data (see [43, 44] for an overview). Meanwhile, smartphone apps that collect usage data provide an unprecedented opportunity to access objective and precise information on smartphone application usage. Hung et al. [45] find that phone call duration and rhythm patterns are predictive of negative emotions, while Saeb et al. [46] predict depressive symptom severity with geographical location and phone usage frequency information. However, as feature engineering with phone app usage data requires considerable time and effort [47], the potential of such data of psychological research is yet to be discovered.

#### Prediction of SWB levels

There have been a few studies aimed at predicting subjective well-being levels, mostly with regression, which obtain modest results. Individual and relational well-being was predicted from social network data [28, 48] and from objective smartphone use data [49]. The reported results are close to the upper bound expected in this task: the meta-analytic correlation between digital traces and psychological well-being has been estimated as $r = 0.37$ across nine studies, including prediction of subjective well-being, emotional distress and depression [28]. The only study that reaches a higher correlation of 0.66 in one of the models [49] does not specify the scales used for measuring SWB; however, interestingly, it finds that while some apps predictably have a negative effect on well-being, others affect it positively.

Diener’s SWLS, to our knowledge, has been predicted in only four studies that use digital traces in a cross-validated setting. In his pioneering study, Kosinski et al. [50] predicted SWLS with linear regression for 2340 Facebook users based on 58K ‘Likes’ – preferences of webpages indicated by the users. The Likes data dimensionality was reduced to top 100 components in a SVD model based on a larger dataset (58K users). The obtained correlation reached $r = 0.17$, whereas empirical test-retest correlation for SWLS was $r = 0.44$.

Collins et al. [51] predicted SWLS with Random Forest Regression and various Facebook features, including demographics, networking data, photos, likes, ground truth Big Five traits of the users, of their significant others and friends, and predicted Big Five as a proxy. The best result for a sample of 1360 users with Big Five features as a proxy reached the Mean Absolute Error (MAE) = 0.162, whereas the model with social network features produced MAE = 0.173 for SWLS. Unfortunately, no other evaluation metrics were reported in this study. Schwartz et al. [52] applied Ridge Regression to predict SWLS of 2198 individuals using their Facebook statuses. Thousands of linguistic features were extracted from the status texts, including 2000 topics obtained with the Latent Dirichlet Allocation topic modeling algorithm, word uni- and bi-grams, LIWC and sentiment lexica. A message-user level cascaded aggregation model was additionally trained on a disjoint dataset, which allowed to improve regression results from Pearson $r = 0.301$ to $r = 0.333$. Facebook status data were also used by Chen et al. [53] to predict SWLS of 2612 users. Features included affect measured by sentiment word usage, 2K topics obtained with topic modeling and 66 LIWC categories. After feature selection with Elastic Net regression, Random Forest model was tested for prediction of an unseen subset. The results reach Root-Mean-Square Error RMSE = 1.30 (0.217 when rescaled to $[0;1]$) and $r = 0.36$.

There is a certain number of studies predicting SWB with app usage data. Some of them rely on self-reported measures of app use [54], while others collect objective data [49, 55]. Correlation in David’s model range from 0.31 to 0.66, however, the research does not specify the scales used for measuring SWB. At the same time, interestingly, it finds that while some apps predictably have a negative effect on well-being, others affect it positively. Gao and colleagues [55] report correlation from 0.34 for male users to 0.66 for female users in the task of predicting SWLS, however, they do not report the full feature set and the contribution of each feature in their best models. Instead, they mention that the most predictive variables are communication apps, certain types of games and the frequency of photo taking. None of these studies mentions cross-validation.

Overall, although the results of subjective well-being prediction are promising, several gaps in the existing research can be identified. First, WHO-5, which is an effective screening tool for depression risk and subjective well-being, has never been studied in a predictive research design. Second, all the studies predicting SWLS are limited to English-speaking populations and respective linguistic features. Moreover, these works only address Facebook digital traces, including profile, texts and likes. Finally, only scarce feature interpretation is reported in the previous studies, and digital trace manifestations of different well-being dimensions have never been compared.

### Our approach

In this study, we set out to predict two different concepts of subjective well-being: one combining affective balance and life satisfaction (measured by SWLS index and further referred to as satisfaction-related SWB) and the other conceptualized as a reflection of mental health (measured by WHO-5 index and further referred to as mental SWB). For predicting well-being values, our task is defined as regression, while for detecting depression risk, we formulate our goal as a binary and trinary classification task. For the latter, we identify the threshold values of WHO-5 by validating them against the scores of the same users on the scales of depression, anxiety and stress, so that the WHO-5 values predicting these scores with the highest sensitivity and specificity are chosen. We perform our prediction of SWB on the texts of private messages, social media and smartphone usage information and perform regression and classification experiments in a cross-validated Machine Learning design. The novelty of the current study lies in the following:

1. We present *the first study so far on predicting subjective well-being measured by WHO-5*;
2. We find out a close association of WHO-5 thresholds with three scales of mental health which is promising in terms of extending our approach to the task of *simultaneous prediction of a range of various mental health risks*.
3. We are the first to *compare satisfaction-based and mental SWB*, analyzing their intersections and differences in terms of predictive features;
4. This is the first study to *combine* language, social media and phone app usage features in well-being research;
5. To our knowledge, our study is the first to address subjective well-being prediction in a *Russian-speaking population* and respective data: the Russian social network VKontakte and texts in the Russian language;
6. We use a dataset of a psychological application users, allowing us to predict *subjective well-being in real-world conditions for a sample with high mental risks*, which has never been done before;

## Materials and methods

### Dataset

Our dataset was collected in collaboration with Humanteq social analytics company, using its DigitalFreud app (DF) – a Russian-language phone application for psychological self-assessment – promoted among Android-based smartphone users through Google Ads. Android was chosen as the basic operational system for data collection, as at the time of the app development and promotion its users constituted the majority (68–76%) [56] of Russian smartphone users who in turn were the app’s target audience and who constituted 57–64% [57] of Russia’s population. Additionally, the app was available to Russian speakers from any country, and although users from the countries other than Russia constituted the minority, none of the samples we further analyze is intended to be representative of Russia.

Data collection via a psychological app of such type was used to access a high-risk population (its high-risk status was confirmed in subsequent comparison of its mean SWB to those in other populations, presented further below). Users were offered to take as many free tests as they wanted (including personality traits, depression, anxiety, stress, cognitive, motivation and SWB tests) and to explicitly consent to the access to their VKontakte profile data and/or smartphone use data. Based on the test results, users were offered psychological feedback and analytics on the use of VKontakte and/or their smartphones. On average, DigitalFreud users chose to fill in 1.5 questionnaires and shared varying subsets of their data, which made the overall dataset quite sparse.

Privacy policy included a clause stating that the data could be used for research. The study was approved by the HSE Ethics Committee; nevertheless, the data were anonymized prior to the analysis. No personal information (i.e. allowing to identify the users) was included in the sample. In particular, all the user profile ids were encrypted.

The initial sample included 2050 accounts of DigitalFreud users who have completed at least one of the two questionnaires of our interest: SWLS [10] or WHO-5 [58]. The vast majority completed either of the tests only once; for those who did it more than once, the earliest score was taken into our dataset.

The following digital traces data were available for the participants:

- DigitalFreud profile data;
- VKontakte user data;
- Phone application data.

Due to data sparsity, our *final sample* used in prediction contains digital traces by 372 users. The procedure of data cleaning that produced this dataset is given in Appendix 1. Thus the dataset is small because the data on well-being combined with personal digital traces is highly difficult to obtain, as it requires both considerable effort from a user on completing the questionnaires, and trust allowing them to share sensitive digital traces. However, our dataset is uniquely tailored to the task of predicting SWB in a high-risk population of mental health app users.

Additionally, there is a *heldout dataset*, which consists of messages written by 572 users, who lack other important features for prediction (demographics, phone app usage) but have text data. The *heldout dataset* is used for preliminary feature selection (see sections *Words*, *Word clusters* below). Before feature selection, texts were tokenized with *happiestfuntokenizing*^1^ and lemmatized it with *pymorphy* [59].

The *phone app dataset* consists of phone application usage data by 992 users who lack other important features for prediction. The *phone app dataset* was used for preliminary phone application categorization and feature engineering.

We also collected a sub-sample of users ($N = 417$), who have completed the WHO-5 and at least one of the following questionnaires evaluating different mental health risks (*mental health dataset*):

1. Depression measured with the Patient Health Questionnaire (PHQ-9) [60];
2. Anxiety measured with the General Anxiety Disorder scale (GAD) [61];
3. Stress measured with the Perceived Stress Scale (PSS) [62, 63].

The *mental health dataset* was used in the WHO-5 classification task to select cutoff thresholds of the classes to be predicted, so the former would be representative of a range of mental health conditions.

#### Self-reported well-being measures

##### Satisfaction-related well-being scale (SWLS)

The SWLS questionnaire was translated to Russian and validated by Ledovaya et al. [64].

The questionnaire contains 5 statements, each characterized by 7-point Likert scale ranging from 1 (strongly agree) to 7 (strongly disagree). The resulting SWLS score ranges from 5 (low satisfaction) to 35 (high satisfaction). The scale has good internal consistency: *α* coefficients ranging from 0.79 to 0.89. Test-retest coefficient, as already mentioned, ranges from 0.54 to 0.84 depending on the time lag between measurements (years or weeks, respectively) [21] and amounts to 0.78 in the Russian language version[64]. In our sample, 1727 accounts have information about the SWLS score.

##### Mental well-being scale (WHO-5)

We use the official Russian-language version of WHO-5 scale developed by WHO itself [58]. Each of WHO-5 items is scored on a 6-point Likert scale ranging from 0 (at no time) to 5 (all of the time). The WHO-5 score ranges from 5 (absence of well-being) to 30 (maximal well-being).The scale has good Internal consistency: *α* coefficients ranging from 0.82 to 0.95 [13]. Test-retest coefficients are available for specific populations only and only in the short run ranging from 0.81 to 0.83 [65, 66]. In our sample, 1791 accounts have information about the WHO-5 score.

##### Mental well-being classes

As mentioned earlier, WHO-5, unlike SWLS, is indicative of a range of mental health conditions [24] and was directly designed to detect one of them [11]. Decisions of mental health, be it screening test results or medical diagnoses, are usually binary and point either at the absence or the presence of a disease. For such tasks scales need to be transformed into sets of discrete classes based on a certain threshold values. Such validated values exist for the original English-language WHO-5 scale (0.28 for major depression and 0.5 for depression). They are recommended for all nations and languages, but in fact have never been tested for the Russian-language population. Meanwhile, it has been shown that cultural differences matter in scale construction [67] and that, specifically, they complicate both mean WHO-5 comparison and threshold comparison across countries [15]. Therefore, we validated several thresholds ourselves. For this, we analyzed the *mental health dataset* of 417 DigitalFreud users who have completed both WHO-5 and one of the three questionnaires – on depression, anxiety and stress – and found the values of WHO-5 index best predictive of the classes of these three scales. This approach was our choice for two reasons:

- the data on clinically diagnosed depression are absent from our dataset;
- the three mentioned scales were validated for the Russian language and thus have been used here as the best available benchmarks.

We tried out different WHO-5 thresholds to reach better sensitivity and specificity in representing the following conditions: PHQ/GAD ≥ 10 for depression and anxiety [68], and PSS ≥ 21 for stress [63]. Additionally, as from our earlier work [69] we know that classes derived from scale reduction might be better predicted in a trinary design in social science NLP tasks, we also experimented with three-class divisions.

Eventually, our analysis resulted in the following cutoff values of the normalized WHO-5 scale:

- Binary cutoff = 0.51 with classes containing 221 and 151 users in the low and high SWB classes, respectively;
- Trinary cutoffs $= [0.35; 0.59]$ with classes containing 111, 158 and 103 users in the low, medium and high SWB classes.

Table 1 illustrates sample statistics for each of the mental health conditions, and specificity and sensitivity in terms of the selected WHO-5 cutoff values.

**Table 1:** Specificity and sensitivity of the selected WHO-5 cutoff values in the *mental health dataset*

Condition	*N* (mental health dataset)	Metric	Binary cutoff (0.51)	Lower trinary cutoff (0.35)	Upper trinary cutoff (0.59)
Depression	344	Sensitivity	0.80	0.49	0.90
		Specificity	0.58	0.87	0.45
Anxiety	309	Sensitivity	0.82	0.53	0.92
		Specificity	0.54	0.83	0.41
Stress	323	Sensitivity	0.85	0.47	0.93
		Specificity	0.66	0.88	0.50

In our high-risk sample of mental health app users, the binary WHO-5 cutoff value 0.51 allows to reach high sensitivity across the analyzed mental health conditions, while preserving moderate specificity. The trinary cutoff values 0.35 and 0.59 allow to obtain low and high mental well-being classes with very high specificity.

#### Digital traces

##### DigitalFreud profile

Account information about the DigitalFreud user includes encrypted DigitalFreud and VKontakte user ids, SWLS and WHO-5 scores, gender, birth year, education, employment and marital status, and date and time of the DigitalFreud app installation.

##### VKontakte user information

Humanteq chooses to match DigitalFreud data with VKontakte data since the latter is the most popular social networking site in Russia. We use the following data obtained with VKontakte application programming interface (API):

1. User Profile data. Although VKontakte API provides access to potentially rich user information, in practice users seldom fill in their profiles, and the data is sparse. As a result, we only use gender, birthdate, and the number of friends and subscriptions in our analysis.
2. Wall posts (text, date and time, information on reposting with the original post contents and encrypted user id, number of reposts, comments and likes) available for 1871 users.
3. Directed private messages (text, date and time, encrypted author and addressee ids) available for 1044 users.

##### Phone application usage

Phone application usage was monitored for one week following the initial consent obtained from the user when she started using DigitalFreud, which was consistent both with the app’s terms of use and the policies of the Android platform. The collected information includes name and package of the application, start time and duration of the application usage in foreground in milliseconds. It is available for 992 users. In a few cases when the users quit the phone app data sharing before the end of the week, the recorded period was shorter.

### Descriptive statistics

**Figure 1:**
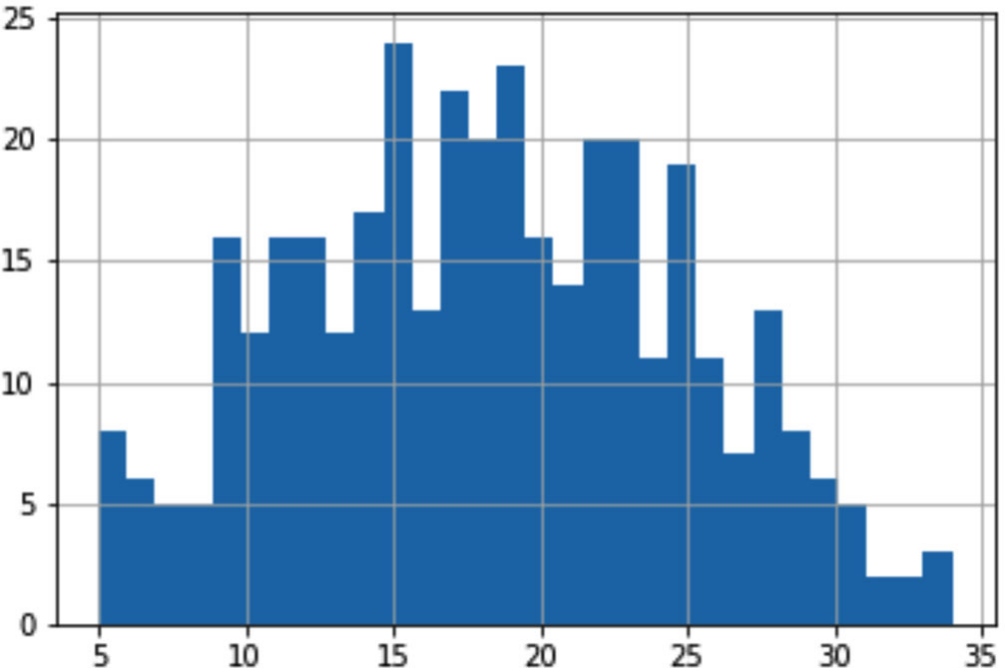
Distribution of SWSL values

**Figure 2:**
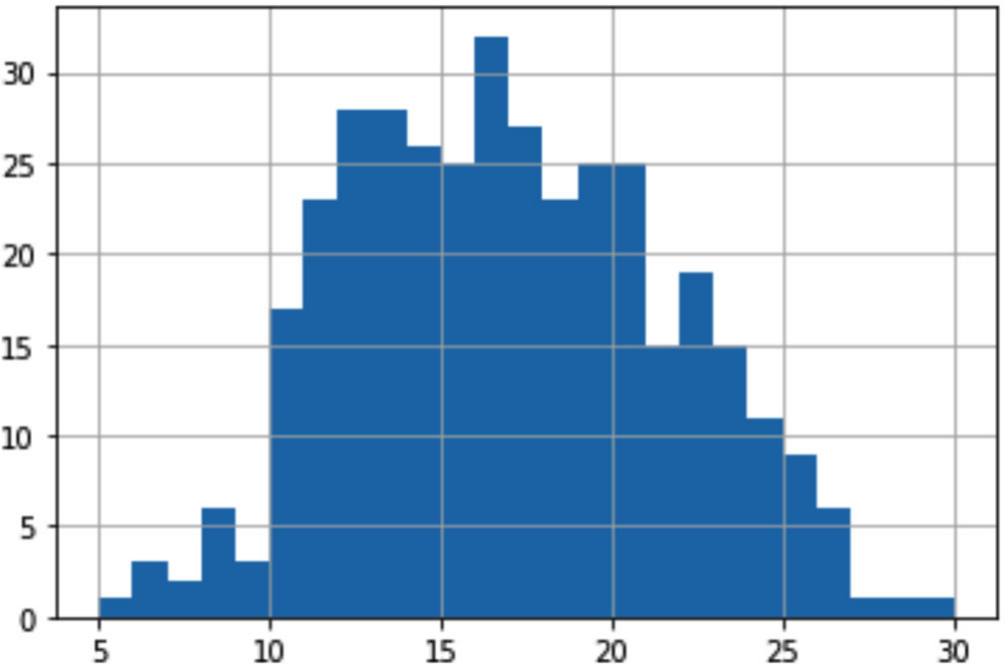
Distribution of WHO-5 values

**Figure 3:**
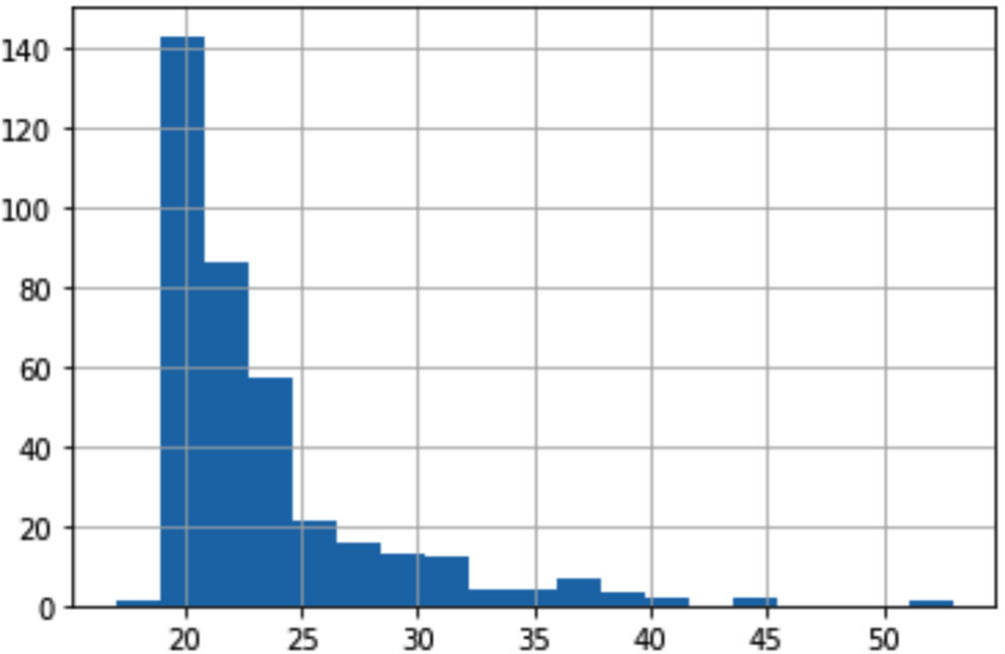
Distribution of Age values

**Figure 4:**
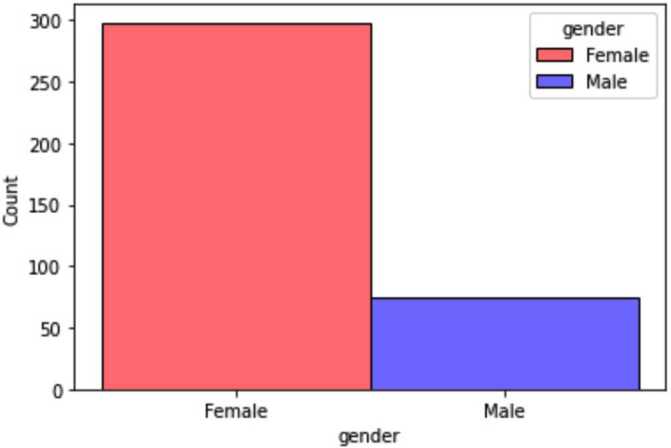
Distribution of Gender values

The main parameters of the descriptive statistics for our *final dataset* of 372 users are given in Tables 2 and 3. Our dataset is predictably skewed towards containing more females (80%) and young people (mean age 23 ± 5 y.o.) against 53% of females and the mean age of 39 y.o. in the general Russian population [70]. However, as it has been mentioned, this sample is not theoretically intended to represent Russia. Consistent with Collins et al [51], we normalize both well-being scores to the ranges between $[0,1]$; to do so, we subtract 5 from both scores, then multiply SWLS values by 1/30, and WHO-5 values by 1/25. Additionally, the distribution of the SWB and demographic data in the *final dataset* is illustrated in Appendix 2, Figs. 1–4.

**Table 2:** Descriptive statistics for subjective well-being, age and gender in the final dataset

	*N*	Range	Mean	Std	Mean (norm)	Std (norm)	Cronbach’s *α*
SLWS	372	5–35	18.30	6.73	0.4433	0.2243	0.8365
WHO-5		5–30	16.51	4.66	0.4604	0.1865	0.8205
Age		18–53	23.06	5.06			
Gender		Male, Female	298 (80%) Female

**Table 3:** Descriptive statistics for the textual and phone app usage features in the final dataset

Data	Sum	Mean	Median	Min	Max
Messages	6739K	18,115	10,948.5	52	131,368
Message alters	53K	143	107.5	2	1029
Message volume (chars)	160,707K	432,009	240,831	671	2,983,231
Posts	7K	19	4	0	1880
Post volume (chars)	857K	2303	84	0	87,708
App Usage (seconds)	1573K	4231	3715.5	24	16,329

SWLS and WHO-5 intercorrelate strongly with $r = 0.568$, $p < 10-32$. The level of internal consistency of both scales is high (Cronbach’s $\alpha > 0.82$).

Both SWB scores in our final sample are consistently lower than in other studies made on other groups of Russians. Thus, WHO-5 score amounts to the average of 0.46 ± 0.187 in our dataset against 0.60 ± 0.191 obtained in a study of Russian Facebook users [71], the only available evaluation of WHO-5 for Russia. Likewise, while the mean SWLS score among our participants is 18.3, a study on a sample close to the general Russian population (mean age 41 y.o. with 54% of women) shows the score of 23.6 [72]. A younger group of Russian students (mean age 20 with 65% of women) which is more similar to our sample scores even higher: 24.4 [73]. The lower SWB levels in our dataset are explained by self-selection of specific individuals to the DigitalFreud app: it naturally attracts users interested in seeking psychological and mental health information and advice, i.e., potentially more likely to have problematic mental health conditions. This is in line with our research goal of studying high-risk populations, of which our sample is an obvious example exactly due to the lower SWB scores.

### Feature engineering

For our task of SWLS and WHO-5 prediction, we construct features of three main types:

- User metadata and overall activity: demographics, DigitalFreud & VKontakte profile statistics, and overall phone app usage statistics;
- Textual, or linguistic features:
  - Words;
  - Sentiment scores;
  - RuLIWC;
  - Word clusters;
- Phone app usage statistics by app category.

Overall, we constructed 660 features for SWLS and 651 for WHO-5. Most features were calculated as counts, ratios or counts by time period directly from the *final dataset*. However, words and word clusters as features were trained on the *heldout dataset* that does not intersect with the *final dataset*. Of these features, only those that correlated with the target variables were selected for the main experiments. In the main experiments, the features were submitted to the regression or classification models, which performed on the *final dataset* that was divided into train, development and test subsets in a 10-fold cross-validation scenario. In this scenario, (1) multiple models were trained on the train set, (2) recursive feature elimination was performed on the development set based on MAE of the models, and (3) final scores for each feature type and each model were computed based on the test set. More details on the main experiment procedure are given in the *Machine Learning Experiments* section.

#### User metadata and overall activity features

There are 40 features describing demographics, overall phone application usage data and the data on the overall activity patterns based on DigitalFreud and VKontakte profiles (see Table 4). The activity-related data include three groups of features: (1) numbers and volumes of personal messages written during one month preceding test completion, (2) numbers of alters, or accounts that a user has a message history with, for every user in each of the 12 months preceding test completion, and (3) weighted differences between the last two months in terms of the message volume and the number of alters. In building phone app usage features, we follow the previous research [74, 75] which identified three- and six-hour periods of online activity to be significant markers of mental illness. In our research, we break phone app usage into three-hour periods of activity. Some features have been excluded from the analysis, due to data saprsity.

**Table 4:** User metadata and overall activity features

Feature name	Description	Number
Age	–	1
Gender	–	1
NVkFriends	N^o^ of friends in VKontakte	1
AllAlters	N^o^ of alters (accounts that a user has a message history with) in the last 12 months	1
Subscriptions	N^o^ of VKontakte page subscriptions	1
Mess_ 1	Total number of messages written in the last 30 days	1
MessChars_ 1	Total size (in characters) of messages written in the last 30 days	1
growth-2to-1weighted	Weighted difference between total size of messages written in the months −1 and −2	1
altersdiff	Weighted difference between numbers of alters in the months −1 and −2	1
AppUsage1Week	Number of active app usage instances in the period of app data sharing time (one week)	1
AllAppTime1Week	Total time of phone app usage in the period of app data sharing time (in seconds)	1
RatioAppTime1Week	Ratio of phone app usage time in the week of app data sharing time	1
AppUsage 0–3, 3–6, 6–9, 9–12, 12–15, 15–18, 18–21, 21–24	Time of phone app usage in 3-hour time periods – each out of the 8 features represents a 3-hour time period	8
AppUsage 0–3, 3–6, 6–9, 9–12, 12–15, 15–18, 18–21, 21–24 Ratio	Time of phone app usage in 3-hour time periods normalized by total app usage time – each out of the 8 features represents a 3-hour time period	8
Alters −1–−12	Numbers of alters in every month (30 days) before the DigitalFreud install time, for months between −1 and −12	12
Total		40

#### Linguistic features

Our extensive analysis of user texts has shown that VKontakte public wall posts are too sparse and include mostly web link content, which does not allow for effective prediction. As a result, we construct all the linguistic features based on private messages written by the users in VKontakte messenger, mostly during one year preceding the installation of DigitalFreud app.

##### Sentiment scores

We use six features representing the proportions of positive and of negative words in the messages created during one month or one year preceding test participation, or in the entire messaging history of a user. Each feature represents the proportion, or l1-normalized frequency, of positive or negative sentiment words written in one of the three time periods (which results in $2\times 3=6$ features). The sentiment words were identified with a closed-vocabulary approach based on the Russian sentiment lexicon RuSentiLex [76].

##### Words

We adopt the open-vocabulary approach to word features predictive of well-being [77]. Given the small size of our final dataset (372 observations), using all the frequent words as features (12K words with frequency ≥ 200) would inevitably result in overfitting. To overcome this and to select a reasonable number of interpretable features, we use the heldout dataset as follows:

- First, a sub-sample of users who have filled both well-being questionnaires was selected from the heldout dataset (396 users);
- Next, we selected 12.5K words occurring more than 200 times in the joint one-year long message collection of all users and calculated their TfIDF scores using 396 individual message collections as 396 texts for such calculation;
- We filtered out words with $p > 0.01$ in the ANOVA tests relating these words to SWLS and WHO-5 values in the heldout dataset, which has resulted in the selection of 165 words for SWLS and 224 words for WHO-5 (see Appendix 3 for the full list). Words belonging to either of these sets (353 words) are used as features for prediction.

##### RuLIWC

For obtaining closed-vocabulary features, we used RuLIWC dictionary – a translation of the most prominent categories of the Linguistic Inquiry and Word Count (LIWC, [78]) performed by Panicheva & Litvinova [79]. RuLIWC consists of eight word categories: Bio, Cognitive, Social, Time, Percept and subcategories of the latter: Feel, Hear, See, with 563–2624 words in each category and 20–303 words in each subcategory. For this research, RuLIWC feature values have been computed as the sums of all the words’ TfIDF values for every user. All the words regardless of their (in)frequency were accounted for.

##### Word clusters

Content features were computed by clustering words with a word2vec semantic model [80] based on the *heldout dataset*. The word2vec model we used had been trained on the web-based Taiga corpus containing over 5 billion words [81] by Kutuzov & Kuzmenko [82], with skipgram algorithm, vector dimensionality = 300, and window size = 2. For clustering, we used 7128 words present in the model vocabulary with frequency ≥ 200 in the *heldout dataset*. Next, we performed KMeans clustering with cosine distance and 300 clusters. As KMeans algorithm is stochastic and may give very different results in different runs, we used the following procedure to obtain reproducible cluster solutions:

- We employed cluster regularization, where the regularization parameter was the sum of p-values of the cluster occurrence correlation with SWLS or WHO-5;^2^ the regularization weights were $[0; 50; 100; 500]$;
- For every weight value, ten random cluster solutions were obtained;
- Based on these solutions, consensus cluster solutions were constructed^3^ with the following thresholds: $[0.25, 0.45, 0.65, 0.75, 0.85]$;
- This resulted in five consensus cluster solutions for every weight value, thus the overall number of solutions totaling to 20.
- In each solution, clusters were additionally augmented with infrequent words in the dataset, every infrequent word being ascribed to the closest cluster. Thus each of 20 solutions was supplemented by a paired solution with augmented clusters.

The clustering results were evaluated on the *heldout dataset* as follows:

- For every cluster solution, only the clusters that correlated with $p < 0.05$ with SWLS or WHO-5 were used as features;
- Each cluster feature was computed as the sum of the respective words’ TfIDF values;
- The resulting features were used for RandomForest regression predicting SWLS and WHO-5 on the *heldout dataset*, with 10-fold train/test cross-validation and recursive feature elimination;
- The best cluster features were chosen by Mean Average Error (MAE) of the regression models trained on the *heldout dataset*; later they were used for prediction on the *final dataset*.

The main parameters of the resulting feature sets are described in Table 5.

**Table 5:** Best word cluster features

	Regularization weight	Consensus clustering threshold	Infrequent words	No of clusters	MAE
SWLS	500	0.45	−	28	0.1704
WHO-5	0	0.45	+	19	0.1525

#### Phone app categories and usage features

The phone app categories and usage features are based on the 1-week phone app usage history shared by the participants. App categories, or types were obtained from the *phone app dataset* data by using 53 app categories generated automatically from 28K app descriptions and by manually uniting them into larger groups as described in [47, 49]. As a result, we identified the following nine app categories: *Game, Education+Productivity, Tools, Entertainment, Personalization, Health+Medical, Social+Communication+Dating, Photography*, covering 21.5K apps, with the rest 6.5K apps having been assigned to *Other*. The main app usage features were calculated as the total time devoted to a certain app category (e.g. *Game*, *Photography* or *Other*) in each of eight three-hour time slots of a day, averaged over all days of a given user ($9*8 = 72$ features), as well as overall time spent for this category in the entire app usage history of an individual (9 features). Next, we constructed several normalized versions of each feature. Namely, we normalized them by the total app usage time in this category, and by the total app usage logged in the current three-hour period. This resulted in $9 + 72*3=225$ features. The phone app category features are exemplified in Table 6.

**Table 6:** Phone app category features

Feature type	N^o^ of features	Example feature name	Description
Total time logged in category by a user	9	GAME	Total time logged in Game apps by a user
Total time logged in category in time period by a user	72	GAME_21-24	Total time logged in Game apps between 21 and 24 h by a user
Total time logged in category in time period/total time logged in category by a user	72	PHOTOGRAPHY_0-3/PHOTOGRAPHY	Ratio of time logged in Photography apps between 0 and 3 AM to total time logged in Photography apps by a user
Total time logged in category in time period/total time logged in time period by a user	72	EDUCATION + PRODUCTIVITY_15-18/15-18	Ratio of time logged in Education+Productivity apps between 15 and 18 h AM to total time logged in apps between 15 and 18 h AM by a user

### Machine learning experiments

We performed specific experiments for each of our two subtasks: prediction of satisfaction-related and mental well-being scales and prediction of the classes in the latter. As we aimed at interpretable results, our main experiments were based on classical regressions. Simultaneously, to make sure that we obtain the best possible prediction quality with the available contemporary methods, we also carried out extensive experiments employing deep learning approaches (described in Appendix 4). However, they yielded inferior results. The two main possible reasons for that are the following (1) our data are hard to obtain, and the obtained data are sparse and loosely intersect between users, which reduces the sample significantly; (2) our message data is hierarchically organized, with numerous alters with whom every participant communicates and numerous messages sent to every alter, while additionally the number of alters and messages highly varies between the participants/alters (see Table 3 above).

Our experiment on prediction of SWLS and WHO-5 scales was performed using a 10-fold cross-validation design with train, development and test sets (298/37/37 users, 80/10/10%). The non-overlapping train, development and test sets were constructed as follows:

1. The sample was shuffled and sorted by the well-being values;
2. The sorted sample was divided into 10 bins containing 37 users each so that $\mathrm{bin}_{i}$ consisted of users with $\mathrm{index} = i + K*37$, where *K* varied in the range $[0; 36]$. Thus every bin was equally distributed in terms of the SWB values.
3. For *i*th cross-validation fold, $\mathrm{bin}_{i}$ was used as the test set, $\mathrm{bin}_{i+1}$ – as the dev set, and the remaining users belonged to the training set.

Our evaluation metrics for *regression* include Mean Absolute Error (MAE), Pearson *r* and *R*2-score. Hyperparameter values were chosen inside the cross-validation loop based on the results obtained from development by MAE values. Recursive Feature Elimination (RFE) was performed based on the development set to identify the informative features in each cross-validation fold. RFE was adopted based on the earlier experiments which had shown the increase in model performance with RFE. Additionally, RFE allows to select a small number of informative features, improving the model interpretability. The selected best hyperparameters and features were used to evaluate the quality of prediction on the test set inside the cross-validation loop. In the end, the evaluation metrics were averaged across all 10 folds.

Predictions of SWLS and WHO-5 scores were performed with seven regression models, including Linear Regression with various regularization techniques, Decision Tree, and two ensemble methods (see Appendix 5). WHO-5 classification was performed with three classification models based on our preliminary experiments (Appendix 6).

*Classification* of individual WHO-5 levels was performed in a *binary* mode with two classes (*low VS high well-being*) and in a trinary mode with three classes (*low VS medium VS extremely high*). The models and hyperparameter values are described in Appendix 6. We report F1-macro and F1-weighted metrics over all the classes, as well as F1 metric for the lowest and the highest classes separately. We additionally report True Positive and False Positive Rates for the low well-being class, as these measures are typically used for screening test of various mental health conditions (cf. [38]).

All the calculations were performed in *python* with *pandas*, *scipy*, and *scikit-learn* libraries.

## Results

### Prediction of well-being scale values

The continuous modeling results for the SWLS and WHO-5 well-being values are presented in Tables 7 and 8, respectively.

**Table 7:** SWLS value prediction results

Features	Best model	Results		
		MAE	Pearson R	R-2
Mean baseline		0.1853	–	–
Median baseline		0.185	–	–
Words	ElasticNet	0.1744	0.3402	0.1022
RuLIWC	DecisionTree	0.182	0.2168	0.0142
AppCats	ElasticNet	0.1762	0.2737	0.0172
Behavior	DecisionTree	0.1785	0.191	0.0195
Clusters	RandomForest	0.1814	0.1709	0.026
*Clusters* + *AppCats* + *Behavior* + *Words*	*ElasticNet*	**0.1698**	**0.4024**	**0.1045**
*Clusters* + *AppCats* + *RuLIWC* + *Behavior* + *Words*	*ElasticNet*	**0.1681**	**0.3776**	**0.1164**

**Table 8:** WHO-5 value prediction results

Features	Best model	Results		
		MAE	Pearson R	R-2
Mean baseline		0.1542	–	–
Median baseline		0.1533	–	–
Words	Lasso	0.1441	0.3179	0.0817
RuLIWC	Lasso	0.1529	0.1276	0.0197
AppCats	ElasticNet	0.1511	0.2172	0.0329
Behavior	DecisionTree	0.1497	0.2463	0.0096
Clusters	Lasso	0.1516	0.1533	0.0241
*Clusters* + *RuLIWC* + *Words*	AdaBoost	**0.1436**	**0.3202**	**0.081**
*AppCats* + *RuLIWC* + *Behavior* + *Words*	*ElasticNet*	**0.1438**	**0.367**	**0.1193**

The results for every individual feature set, and for the best feature sets in terms of every evaluation metric are included; the best results are highlighted in bold. The full results for all the feature set combinations are presented in Appendices 7, 8.

Overall, the best feature set is words written by the users in messages, and the best model is ElasticNet.

### Prediction of WHO-5 classes

The main classification results for the WHO-5 well-being are presented in Table 9. The full WHO-5 classification results are presented in Appendix 9.

**Table 9:** Best WHO-5 classification results

Classifi cation	Thre-shold	N (Classes)	Best model	Best features	F1-macro	F1-weigh-ted	F1-low	F1-high	True Positive Rate (low)	False Positive Rate (low)
Binary	0.51	221/151	Ada-Boost	Words + RuLIWC + AppCats	0.692	0.706	0.768	0.616	0.792	0.404
Binary majority baseline					0.378	0.456	0.373	0	1.0	1.0
Trinary	0.35/0.59	111/158/103	Ada-Boost	Clusters + RuLIWC + Words	0.483	0.493	0.502	0.433	0.450	0.161
Trinary majority baseline					0.199	0.253	–	–	0.0	0.0

### Significant features

The features in the best performing continuous models of satisfaction-related well-being (SWLS) and mental well-being (WHO-5) scales are illustrated in Tables 10 and 11. Only the features which were selected by RFE in at least five out of ten cross-validation folders are included; the features significant in both SWLS and WHO-5 regression are highlighted in bold. All the significant features are listed in Appendices 10, 11.

**Table 10:** Predictive features in SWLS scale. Slang, misspellings and unconventional word forms are shown with an asterisk (*). Errors in lemmatization are enclosed in brackets

Feature type	Feature	Translation/Description	Coefficient
Words	спать_[NOUN]	sleep_VERB	41,086
	интим_NOUN	intimacy_NOUN (suggestive of ‘intercourse’)	−44,937
	орг_NOUN*	org(aniser)_NOUN	23,978
	дропнуть_VERB*	quit_VERB	−64,677
	тратиться_VERB	spend_VERB	−24,593
	отл_UNKN*	fine_UNKN	34,184
	пояснение_NOUN	explanation_NOUN	−22,499
	стебать_VERB*	bully_VERB (rude)	−28,898
	[вифя]_NOUN*	wifi_NOUN	−48,114
	спойлерить_VERB*	spoil_VERB	−48,530
	ооохнуть_VERB*	gasp_VERB	−44,864
	милый_COMP	nice_COMPARATIVE	56,128
	[пиздёжа]_NOUN*	lie_NOUN (rude)	−22,727
	обжечь_VERB	burn_VERB	−40,019
Sentiment	*Negative_month*	negative sentiment in the last month	−29
Activity	*AppUsage9-12Ratio*	Ratio of phone app usage time between 9 and 12 AM normalized by total app usage time	10
	AppUsage0-3Ratio	Ratio of phone app usage time between 0 and 3 AM normalized by total app usage time	−8
AppCats	SOCIAL + COMMUNICATION + DATING_0-3/SOCIAL + COMMUNICATION + DATING	Ratio of time logged in Social + Communication + Dating apps between 0 and 3 AM to total time logged in Social + Communication + Dating apps	11
	PHOTOGRAPHY_18-21/18-21	Ratio of time logged in Photography apps between 18 and 21 h PM to total time logged in apps between 18 and 21 h PM	8

**Table 11:** Predictive features in WHO-5 scale

Feature type	Feature	Translation/Description	Coefficient
AppCats	GAME_3-6/GAME	Ratio of time logged in Game apps between 3 and 6 h AM to total time logged in Game apps	−5
	ENTERTAINMENT_3-6/ENTERTAINMENT	Ratio of time logged in Entertainment apps between 3 and 6 h AM to total time logged in Entertainment apps	4
	HEALTH+MEDICAL_3-6/HEALTH+MEDICAL	Ratio of time logged in Health + Medical apps between 3 and 6 h AM to total time logged in Health + Medical apps	3
	PERSONALIZATION_0-3/0-3	Ratio of time logged in Personalization apps between 0 and 3 h AM to total time logged in apps between 0 and 3 h AM	−4
	EDUCATION + PRODUCTIVITY_9-12/EDUCATION + PRODUCTIVITY	Ratio of time logged in Education + Productivity apps between 9 and 12 h AM to total time logged in Education + Productivity apps	−3
	TOOLS_18-21/18-21	Ratio of time logged in Tools apps between 18 and 21 h PM to total time logged in apps between 18 and 21 h PM	−3
	SOCIAL + COMMUNICATION + DATING_3-6/SOCIAL + COMMUNICATION + DATING	Ratio of time logged in Social + Communication + Dating apps between 3 and 6 AM to total time logged in Social + Communication + Dating app	7
	GAME_9-12/GAME	Ratio of time logged in Game apps between 9 and 12 h AM to total time logged in Game apps	2
	OTHER_3-6/OTHER	Ratio of time logged in Other apps between 3 and 6 h AM to total time logged in Other apps	−2
	ENTERTAINMENT_9-12/ENTERTAINMENT	Ratio of time logged in Entertainment apps between 9 and 12 h AM to total time logged in Entertainment apps	2
	PHOTOGRAPHY_0-3/PHOTOGRAPHY	Ratio of time logged in Photography apps between 0 and 3 h AM to total time logged in Photography apps	−2
	EDUCATION + PRODUCTIVITY_21-24/EDUCATION + PRODUCTIVITY	Ratio of time logged in Education + Productivity apps between 21 and 24 h PM to total time logged in ducation + Productivity apps	−2
RuLIWC	Bio_RuLIWC	Words related to Biological processes in RuLIWC	−20
Words	(face-blowing-a-kiss_emoji)_UNKN	(face-blowing-a-kiss_emoji)	35
	но_CONJ	but_CONJ	−16
Activity	*AppUsage9-12Ratio*	Ratio of phone app usage time between 9 and 12 AM normalized by total app usage time	7
Sentiment	*Negative_month*	negative sentiment in the last month	−33
	Negative_year	negative sentiment in the last year	−29
	Negative_all	negative sentiment in overall messages	−23

## Discussion

In this paper, we have introduced a novel task of predicting mental well-being measured by WHO-5 index, as compared to traditionally studied satisfaction-related SWLS, with digital traces, and performed it in both continuous modeling and classification designs. In the latter, we have shown that the selected WHO-5 thresholds are representative of a range of three mental well-being-related conditions (depression, anxiety and stress) with high sensitivity and specificity. Furthermore, the results obtained in mental well-being classification are highly promising (0.792 True Positive Rate and 0.404 False Positive Rate) in the binary task with our highly sensitive threshold. This threshold is very close to the one recommended by WHO for moderate depression screening (0.51 against 0.50). The classification result itself is similar to the performance of the best existing models that predict other mental conditions with digital traces [30, 38]. Likewise, our results of SWLS and WHO-5 scale prediction, with Pearson $r = 0.402$ and 0.367, respectively, improve the state-of-the-art metrics reported previously in similar tasks with cross-validation designs [51, 53]. Since, as mentioned earlier, prediction of internal states with observable behaviors has its limitations [29, 30], the obtained correlation may be considered high. As a result, we obtain a model which is highly sensitive and sufficiently specific for identifying low levels of subjective well-being requiring intervention in a high-risk population of mental health application users. Our model is unique not only in its accurate prediction of WHO-5 classes that have a proven ability of depression risk detection, but also in its potential to develop into a tool for broader screening for mental health risks, not limited to specific conditions reported in previous studies (see [28, 30, 48] for an overview).

We have performed a unique comparison of regression models predicting both SWLS and WHO-5 indices on the same sample. Our best models for both indices show similar performance in terms of correlation and R2 metrics, but WHO-5 is predicted better in terms of MAE across all feature combinations; however, this is likely an outcome of different distributions of SWLS and WHO-5 in our sample (see Fig. 1, 2, Table 1 above).

Our design also allows us to compare the features predictive of life satisfaction-related SWB and mental SWB. Although our experiments have revealed only two highly predictive features that are common for both SWLS and WHO-5, they are highly interpretable in terms of psychological theory. These two metrics are (1) phone app usage time between 9 and 12 AM normalized by total app usage time, and (2) negative sentiment expressed in private messages in the last month, which have positive and negative coefficients, respectively, in both SWLS and WHO-5 tasks. Both of these findings confirm previous results obtained in various populations: participants affected by depression and other low SWB conditions have been found less likely than average individuals to participate in online activities in the morning hours around 9–10 AM [74, 75], while their circadian rhythms have been often disrupted [7]. Such disruption is what usually accompanies insomnia or hypersomnia, a symptom of the major depressive disorder listed in DSM-5 [83], the Diagnostic and Statistical Manual of Mental Disorders developed by the American Psychological Association.

Negative sentiment has been shown to correlate negatively with life satisfaction [34, 53, 84] and subjective well-being [71]. Negative sentiment in written or oral speech may also sometimes, although not always, be a manifestation of depressed mood, another symptom of depressive disorder according to DMS-5.

Thus, these two highly predictive features intersecting in both SWLS and WHO-5 prediction models can indicate different degrees of SWB: from simple dissatisfaction with life, circumstances or personal achievements (relevant for SWLS), to a deterioration in mental or physical condition and serious symptoms of the depressive spectrum (relevant for WHO-5). They can be recommended for use across various SWB-prediction tasks.

Predictors unique for satisfaction-related well-being are much more dominated by verbal features related to affect-laden psychological and social content. They are often obscene lexemes, but also represent both negative and positive sentiment polarities (*quit_VERB, spend_ VERB, fine_UNKN, explanation_NOUN, bully_VERB, spoil_VERB, gasp_ VERB, nice_COMPARATIVE*). Association of positive lexica with SWB is consistent with Weismayer [85], who also finds negative relation of SWB with lexica expressing anger and fear. Some of our predictive words are likely to express these emotions (e.g. *bully [rude]*, *burn*, *lie [rude]*, *gasp*). Also, these lexica fit well with some of the ontologies developed for depression detection [45]. Prevalence of lexical features among SWLS predictors suggests that this index, indeed, captures subjective perception of well-being rather than symptoms of mental disorders, such as depression.

On the contrary, in mental well-being level prediction, phone app usage features take a clear lead, especially those related to the ratio of nighttime app usage (3–6 AM). Additionally, lexica related to biological processes are also a distinctive marker of low WHO-5 levels. All this aligns well with the primary goal of WHO-5 to reveal depression and its proved ability to differentiate between problematic mental health states and high levels of mental health-related well-being. Specifically, app usage rhythms and biological lexica are likely to be manifestations of such depression symptoms as increase or decrease in either weight or appetite, insomnia or hypersomnia, and fatigue or loss of energy [86]. At the same time, they can be markers of a poor physical condition, which is also detected by WHO-5 [18].

Finally, the significance of negative sentiment in the long periods of messaging (1 year and longer) for WHO-5 levels suggests that mental SWB measured by this index might in fact have a more stable behavioral pattern than SWLS. However, there is also a possibility that the stable component of SWLS is underrepresented in our features or subjects. Simultaneously, it may be that not only SWLS (as shown in [21]), but also WHO-5 contains both stable and transient components that may be explained by different factors. While the temporal stability of SWB may be expected to be related to constant individual features, such as presence of a chronic disease, SWB volatility, on the contrary, should be explained by short-term mood fluctuations and long-term meaningful changes in life, such as those listed in the introduction. Individual predictors of SWB stability and volatility may differ for SWLS and WHO-5, and it may happen that in our sample the feature set is skewed in favor of WHO-5 stability factors. In any case, our analysis of the overlapping and the differing predictors for WHO-5 and SWLS shows that satisfaction-related SWB and mental SWB share some of their transient factors rather than stable ones. These preliminary observations of the temporal dimension of SWB set a promising direction for future research.

## Conclusions

The growing interest in tracking human mental states and in the development of mindfulness leads to the growth of applications that screen or even diagnose mental conditions and offer solutions for their correction, including those based on objective data. Our research has shown that it is possible to create machine learning models based on interpretable traits and predict various aspects of subjective well-being at the state-of-the-art level.

In doing so, we have performed *the first study on predicting subjective well-being measured by WHO-5*. We have demonstrated that certain WHO-5 level thresholds are indicative of a range of mental health conditions prevalent in a sample characterized by high risk of mental health problems. We have obtained promising results in classification of mental SWB into classes constructed based on these thresholds. This approach has allowed us to identify individuals affected by low subjective well-being with high recall and reasonable false positive rates, based on their digital traces.

Our study is also *the first to compare prediction performance and predictive features of mental SWB and satisfaction-related SWB*. We show that several predictors are shared by well-being measured by both WHO-5 and SWLS, and these digital traces are bluntly indicative of overall (un)well-being. At the same time, digital traces distinguishing between WHO-5 and SWLS are closely related to the conceptual difference between these two indices: while SWLS is characterized by expressions denoting affect-laden psychological and social content, WHO-5 levels are manifested in objective features reflecting physiological functioning and somatic conditions, i.e., lexica related to biological processes and circadian rhythm-related ratios of phone app usage.

To our knowledge, this is *the first approach to subjective well-being prediction in a Russian-speaking population*, and *the first to combine language, social network and phone app usage features* in well-being research. By leveraging phone app usage logs, profile and message data from the Russian social network VKontakte, we have been able to improve prediction of satisfaction-related SWB (SWLS) and propose a first predictive model for mental SWB (WHO-5). At the same time, as our sample has been very small and limited to a high-risk population, the study needs replication on larger samples representative of wider social and psychological groups. The major obstacle to this is that VKontakte private message data are no longer available for any type of download, while other social media are even more restrictive. Development of public policies and regulations encouraging private data-collecting companies to share portions of their data for public good purposes is highly recommended.

## Data Availability

The data that support the findings of this study belong to the Humanteq company and were collected under specific terms of use. When onboarding in the Digital Freud app, users agreed to a privacy policy that explicitly prohibited data transfer to third parties, largely because the amount of data for each user does not allow completely anonymizing the dataset and contains sensitive information. Therefore restrictions apply to the availability of these data, which is why they are not publicly available. A fraction of the data can however be obtained from the authors upon reasonable request and with permission of the Humanteq company. The code for data analysis is available at https://github.com/hse-scila/bewell.

## Appendix 1: Data preprocessing

The dataset was cleaned:

- First, birth year and gender were identified from DF and VK profile data. We removed data where age or gender were not available, or contradicted between DF and VK;
- We only selected users having non-zero information on phone app usage, number of VK friends and at least 100 characters written in messages in the month immediately prior to DF installation. This left us with user 446 accounts;
- We removed duplicates of VK and DF id from the data, giving priority to the data profiles which included more information filled in, and to profiles which were characterized by a later DF install time.
- The resulting final dataset contained 372 unique users with SWLS, WHO-5 and digital traces information.

## Appendix 2: Distribution of the subjective well-being and demographic data in the final dataset

See Figs. 1–4.

### 2.1 Demographic data description

Total sparse data sample includes information about 1960 users. 17.6% of them (344) do not provide information about the country. The rest of the sample (1616 users) contains 84.4% of Russian users, 5% – Belarus, 2.4% – Ukraine, 1.9% – Kazakhstan, 1.1% – USA. We also have users from the countries below, but their frequencies are not higher than 1%: Japan, South Korea, Moldova, Germany, Great Britain, Canada, Finland, Kyrgyzstan, Italy, Argentina, Norway, Israel, Cyprus, China, Vatican, Honduras, India, Serbia, Latvia, Liechtenstein, Iceland, Uzbekistan, Hungary, Georgia, Denmark, France, Ivory Coast, Cook Islands, Estonia, Australia, Romania, Netherlands, American Samoa, Albania, Gambia. Information about the city of the current sample is absent in 30% of cases (578), but the distribution of ten most common cities for the rest of the users (1382) is described in Table 12.

**Table 12:** Distribution of the most common cities identified in the overall data sample

City	Percents
Moscow	47.4
St. Petersburg	36.9
Yekaterinburg	8
Kazan	6.2
Minsk	5.7
Chelyabinsk	5.7
Novosibirsk	5.7
Nizhny Novgorod	5
Krasnodar	4.7
Rostov-on-Don	4.2

The final dataset contains 372 users: 15% of them (57) do not provide information about the country. The rest of the sample (315 users) contains 92% of Russian users, 2.5% – Belarus, and 1.3% – Ukraine. There are also users from the countries below (each has less than 1%): Kazakhstan, USA, Latvia, China, Finland, Norway, Japan, Hungary, South Korea, Canada. Information about the city of the current sample is absent in 26% of cases (98), but the distribution of ten most common cities for the rest of the users (274) is described in Table 13.

**Table 13:** Distribution of the most common cities identified in the final dataset

City	Percents
Moscow	41.6
St. Petersburg	31.9
Yekaterinburg	8
Nizhny Novgorod	5.3
Voronezh	4.4
Chelyabinsk	4.4
Vladivostok	4.4
Tyumen	3.5
Kirov	3.5
Yaroslavl	3.5

## Appendix 3: Word features

See Table 14.

**Table 14:** Total list of words used as features for the SWLS and WHO-5 prediction

SWLS	WHO-5
1000_NUMB	!_PNCT
22_NUMB	2000_NUMB
https://ru.wikipedia.org/wiki/_LATN	2500_NUMB
t_LATN	r_LATN
адрес_NOUN	аааа_NOUN
апрель_ NOUN	ааааа_NOUN
ахуесть_VERB	ааааааааааааааааа_NOUN
ахуеть_ VERB	адрес_NOUN
ахуй_NOUN	анимешник_NOUN
бабочка_NOUN	арми_ NOUN
баня_NOUN	ахахахи_NOUN
бгод_NOUN	ахахахха_NOUN
бесить_ VERB	байка_ NOUN
бланк_NOUN	бантан_ NOUN
бля_INTJ	блестеть_ VERB
блядь_INTJ	блч_UNKN
блятба_ NOUN	блять_NOUN
блять_NOUN	бляяяяять_VERB
бляять_ GRND	бляяяяяять_GRND
большой_ADJ	борис_NOUN
борис_NOUN	будто_CONJ
будто_CONJ	валя_NOUN
бухать_ GRND	вежливый_ADJ
василий_NOUN	вифя_NOUN
ващий_ADJ	вообще_ ADV
вечно_ADV	воооот_ NOUN
водный_ADJ	воскресение_ NOUN
воскресение_NOUN	впервые_ADV
впустить_VERB	впустить_VERB
выглянуть_VERB	вскрыться_VERB
графика_NOUN	выпилиться_VERB
грубый_ADJ	выставить_VERB
даж_UNKN	выступить_VERB
делаться_VERB	глупенький_ADJ
день_NOUN	горе_NOUN
добрый_ADJ	гуглить_VERB
договориться_VERB	даун_NOUN
долбиться_VERB	дельфин_NOUN
е_NOUN	демон_NOUN
ебал_NOUN	дерьмо_NOUN
ебануться_VERB	джон_NOUN
ебать_VERB	джуна_NOUN
еби_UNKN	дилемма_NOUN
еблана_ NOUN	добровольно_ADV
ебу_UNKN	добрый_ADJ
ет_UNKN	доказательство_NOUN
жарко_ADV	дразнить_VERB
жить_VERB	дропнуть_VERB
заебал_ NOUN	ебаный_ADJ
заебок_ NOUN	ебу_UNKN
закрыться_VERB	ет_ UNKN
замечание_NOUN	ж_CONJ
запасный_ADJ	жестокий_ADJ
запрещать_VERB	животный_ADJ
знач_NOUN	загуглила_NOUN
именно_ PRCL	заезжать_VERB
комиссия_NOUN	заехать_VERB
корея_NOUN	замуж_ADV
кофеёк_ NOUN	запереть_VERB
критерий_NOUN	заржать_VERB
лана_NOUN	засиживаться_VERB
лариса_ NOUN	звонить_VERB
лень_NOUN	звёздочка_NOUN
ложь_NOUN	инглихой_COMP
лях_NOUN	интим_NOUN
маман_NOUN	истинный_ADJ
мамаша_ NOUN	как_CONJ
маркетинг_NOUN	кальян_NOUN
маркус_ NOUN	камбэк_NOUN
милах_NOUN	капёс_NOUN
мразь_NOUN	кб_NOUN
мудак_NOUN	коль_CONJ
мёд_NOUN	комикс_NOUN
набрать_VERB	кореец_NOUN
научный_ADJ	корея_NOUN
нах_UNKN	косплей_NOUN
нахуй_NOUN	кпоп_NOUN
нееет_UNKN	ладить_ VERB
ненавидеть_VERB	листочек_NOUN
несмотря_PREP	лосиный_ADJ
неудобный_ADJ	магнитный_ADJ
никто_NPRO	милах_NOUN
нихуй_NOUN	милый_COMP
обжечь_ VERB	монст_NOUN
окончание_NOUN	мразь_NOUN
орало_NOUN	мррррра_NOUN
орг_NOUN	мутный_ADJ
организация_NOUN	мфц_UNKN
отвлечь_VERB	мэн_NOUN
отвратительный_ADJ	набрать_VERB
отл_UNKN	наверна_NOUN
отлично_ADV	наехать_VERB
отсталый_ADJ	намджуна_NOUN
передать_VERB	наорать_VERB
петух_NOUN	настолько_ADV
пизда_NOUN	неинтересно_ADV
пиздец_ NOUN	неловко_ADV
пиздуть_VERB	ненавидеть_ VERB
пиздёжа_NOUN	несчастный_ADJ
подробный_ADJ	нету_PRED
поебать_VERB	неудобно_ADV
пока_ADV	никогда_ADV
показатель_NOUN	но_CONJ
получить_VERB	ноооо_NOUN
пользователь_ NOUN	ноут_NOUN
помереть_VERB	обидный_ADJ
помеха_ NOUN	облизывать_VERB
потерять_PRTS	объяснять_VERB
похуй_NOUN	объёмный_ADJ
пояснение_NOUN	он_ NPRO
предать_VERB	оооо_ NOUN
предсказуемый_ADJ	ооооооо_NOUN
признак_NOUN	оооохнуть_VERB
приобрести_VERB	ооохнуть_VERB
припереться_VERB	орало_NOUN
прогуливать_VERB	останавливать_VERB
прогулять_VERB	отбирать_ VERB
равно_CONJ	отвлечься_VERB
разом_ADV	отвратительный_ADJ
разреветься_VERB	отвратный_ADJ
разрывать_VERB	отлично_ADV
рамка_NOUN	офф_UNKN
растеряться_VERB	ох_INTJ
результат_NOUN	паника_NOUN
рил_NOUN	педик_NOUN
руководитель_ NOUN	переключить_VERB
рушить_ VERB	переписывать_ VERB
рэп_NOUN	пересматривать_ VERB
свалить_VERB	пиздец_NOUN
скот_NOUN	пират_NOUN
скучно_ ADV	писаться_VERB
смеяться_VERB	подъехать_VERB
сосуд_NOUN	поебать_VERB
спока_NOUN	пожениться_VERB
спорый_ADJ	покинуть_VERB
ссылка_ NOUN	помнить_VERB
стебать_VERB	поплакать_VERB
сук_NOUN	порешать_VERB
съебывать_VERB	поступок_ NOUN
тиндёр_ NOUN	потерянный_ADJ
тратиться_VERB	потерять_PRTS
труп_NOUN	поттер_NOUN
трус_NOUN	пошло_ADV
тэхен_NOUN	ппц_UNKN
ущербный_ADJ	предатель_ NOUN
факультет_NOUN	предать_VERB
херить_ VERB	привет_NOUN
хит_NOUN	пригонять_VERB
хм_INTJ	приобнять_VERB
хрень_NOUN	продумать_VERB
хуй_NOUN	прописать_VERB
хуйня_NOUN	псих_NOUN
хула_NOUN	психануть_VERB
хы_UNKN	пытаться_VERB
цель_NOUN	пялить_VERB
через_PREP	работа_ NOUN
шава_NOUN	разреветься_VERB
шеф_NOUN	разрывать_VERB
шлюшка_ NOUN	расплатиться_VERB
шуга_NOUN	расстроить_PRTF
эт_UNKN	растягивать_VERB
эх_INTJ	реветь_VERB
я_NPRO	репер_NOUN
(glowing-star_emoji)_UNKN	репетиция_NOUN
(thinking-face_emoji)_UNKN	риал_NOUN
	рил_NOUN
	рушить_VERB
	саба_NOUN
	сам_ADJ
	свалить_VERB
	серега_ NOUN
	серия_NOUN
	слеза_NOUN
	слишком_ADV
	смеяться_VERB
	спасать_VERB
	спать_NOUN
	спойлерить_VERB
	спорый_ADJ
	ссора_NOUN
	старший_NOUN
	стебать_VERB
	страдать_VERB
	страшно_ADV
	стремный_ADJ
	съездить_VERB
	таак_NOUN
	тони_NOUN
	тренировка_NOUN
	труп_NOUN
	тц_UNKN
	тэхен_NOUN
	убивать_VERB
	удовлетворение_NOUN
	умирать_VERB
	умыться_VERB
	упад_NOUN
	фандом_ NOUN
	ханна_NOUN
	хардкор_NOUN
	хдд_UNKN
	хл_UNKN
	хм_INTJ
	хорошо_ADV
	хотя_CONJ
	худой_COMP
	червь_NOUN
	через_PREP
	чертовый_ADJ
	чонгук_ NOUN
	чувство_NOUN
	чудом_ADV
	чуть_ADV
	шлюшка_NOUN
	шов_NOUN
	шуга_NOUN
	ь_UNKN
	это_NPRO
	этот_ADJ
	юнга_NOUN
	я_NPRO
	(medium-light-skin-tone_emoji)_UNKN
	(face-blowing-a-kiss_emoji)_UNKN
	(drooling-face_emoji)_UNKN

## Appendix 4: Preliminary deep learning experiments

### 4.1 RuBERT

First, we performed experiments with RuBERT models [Kuratov & Arkhipov 2019] based on post and message data. The difficulty in applying BERT-like models in our textual data lies in the fact that BERT model input is limited with max. 512 sub-tokens; at the same time, posts and messages in Vkontakte can be much longer and don’t have a small character limit (as it is, for example, in Twitter). This results in 2 issues, which have to be solved to apply RuBERT to our data:

- input sequences should be truncated to 512 sub-tokens maximum;
- input sequences by the same user should be aggregated.

Solving these issues is not a trivial task for VKontakte posts and messages for the following reasons:

- Posts and messages have different length, they can be much longer than 512 sub-tokens;
- The numbers of posts, messages and message alters for every user vary a lot;
- The rhythm of posting/messaging varies a lot for every user: while active during one month, a user can have no posts or messages written in the previous 6 months;

Post/message information aggregation involves pooling of the individual RuBERT model results, which means basically averaging information between the range of posts/messages by a user, whereas a lot of information is lost. Due to these reasons, we performed most of our RuBERT-based experiments with posts, which, due to their smaller numbers, are easier to aggregate in the RuBERT models. We used data by 902 users with at least 10 posts. We fed each post into one of the RuBERT models [Kuratov & Arkhipov 2019] after truncation. After the RuBERT model, we used a variety of additional layers. Regression was always performed by the final Dense layer. The experiment hyperparameters included the following:

- Using RuBERT as an embedding layer or fine-tuning it for the regression task;
- The models included: RuBERT, Conversational RuBERT, Sentence RuBERT;
- We included all users (902), and those having at least 50 messages (222);
- We used the train/dev/test 5-fold cross validation;
- We included up to 64 posts by each user truncated to 128 sub-tokens each;
- We also aggregated the latest posts by each user and truncated the result to 512 sub-tokens;
- We used the full RuBERT output or the last ‘class’ token;

The layers after the RuBERT models were:

- Dense;
- LSTM+Dense;
- LSTM+Dense+Dense;
- LSTM+LSTM+Dense;

In LSTM layers, the number of units ranged in $[8; 16; 64; 100]$; Dropout rate $= [0., 0.1, 0.3, 0.5]$, optimizers = [RMSprop, Adagrad], learningrate $= [0.0001, 0.001, 0.005, 0.01, 0.05]$, activation = [linear, relu, sigmoid], batch size = 128, epochs = 100, metrics = [mse], early stopping on validation MSE with patience = 10. Unfortunately, the results of these experiments were highly unstable, with MSE values not exceeding the dummy baseline (standard deviation of the sample), and Pearson R reaching 0.1.

#### 4.1.1 Sentiment analysis with RuSentiment BERT

As it was mentioned before, Chen et al. [2016] used sentiment analysis to predict SWLS; we also performed experiments with user messages to assess sentiment. The idea is that distribution over sentiment classes can be used as features for predicting subjective well-being levels. Their many different approaches to classifying messages by sentiment. One of them is to use word dictionaries with sentiment marks. However, it has two important disadvantages: the sentiment of a word can be changed by the context of its use; it is not clear which label should we assign to messages with many words of different sentiment (especially if they are distributed evenly inside the message). These disadvantages lead us to use another common approach for sentiment classification. We used a pre-trained neural network. We found an open-source model with BERT architecture [Devlin J. et al., 2018] which was trained to define the sentiment of VKontakte posts. To be more precise, this model is a result of fine-tuning multilingual BERT with linear head on top using the RuSentiment dataset [Rogers A. et al., 2018] on five classes (“neutral”, “negative”, “positive”, “speech act”, “skip”) classification task.

Using a held-out dataset we subsample users who provide access to their messages. We created a dataset with around 400 users containing messages which were written by them in the last three months before they achieved a WHO score. By providing an exploratory data analysis we found that 10 per cent of users have less than 30 messages, so we cut off these samples. The resulting dataset has 354 samples where each user on average has 4,719 messages (median: 2,415). We normalize the frequency of each sentiment class using the overall number of messages corresponding to a user.

First, we check the correlation between the sentiment classes and WHO score. Table 15 shows that there is no strong correlation.

**Table 15:** Correlation between sentiment class and WHO score

Sentiment class	Correlation with WHO score
negative	−0.14921
positive	0.024321
neutral	0.09399
speech	**0.152864**
skip	−0.114221

We also construct a pipeline with a regression model on top of this frequency distribution with different feature combinations, but the models do not show promising results (Table 16).

**Table 16:** Results for linear regression model with sentiment class frequency features. Mean absolute error and Pearson correlation

Sentiment classes combinations	Mean absolute error	Pearson correlation
negative, positive	0.1434	0.1243
negative, neutral, positive	**0.1445**	0.1265
negative, neutral, positive, skip, speech	0.1447	**0.136**

We assume that achieved results can be explained in the following way. The domain of VKontakte message texts can be different from the domain of VKontakte post text. First, because posts can be interpreted as a complete (finite) phrase, but not a message, which should be interpreted inside the dialogue context. A separated message can have not enough information to classify its sentiment. The absence of dialogue boundaries (when a user starts one dialogue session and finishes it inside a long thread) does not allow us to reconstruct context for a message, which possibly can help to gain a more accurate sentiment classification.

## Appendix 5: Models and hyperparameters used for SWLS and WHO-5 regression

See Table 17.

**Table 17:** Models and hyperparameters used for SWLS and WHO-5 regression

Model	Hyperparameters
AdaBoostRegressor	loss’: [‘linear’, ‘square’, ‘exponential’], ‘n_estimators’: [10,100]
DecisionTreeRegressor	criterion’: [‘mae’], ‘max_depth’: [2,3], ‘min_samples_leaf’: [2], ‘max_leaf_nodes’: [3], ‘splitter’: [‘best’], ‘min_samples_split’: [2], ‘max_features’: [‘auto’]
ElasticNet	alpha’: [100,10,1,0.1,0.01,0.001,0.0001], ‘normalize’: [False, True], ‘selection’: [‘cyclic’, ‘random’], ‘max_ iter’: [500,1000], ‘l1_ratio’: [0.25,0.5,0.75]
Lasso	alpha’: [100,10,1,0.1,0.01,0.001,0.0001], ‘normalize’: [False, True], ‘selection’: [‘cyclic’, ‘random’],’max_iter’: [500,1000,2000]
LinearRegression	normalize’: [False, True]
RandomForestRegressor	n_estimators’: [2,5,10,20], ‘max_depth’: [2,3], ‘min_samples_split’: [2], ‘min_samples_leaf’: [1], ‘max_ features’: [‘auto’]
Ridge	alpha’: [100,10,1,0.1,0.01,0.001,0.0001], ‘normalize’: [False, True]

## Appendix 6: Models and hyperparameters used for WHO-5 classification

See Table 18.

**Table 18:** Models and hyperparameters used for WHO-5 classification

Model	Hyperparameters
AdaBoostClassifier	“algorithm”: [“SAMME.R”]
DecisionTreeClassifier	“criterion”: [“gini”, “entropy”], “max_depth”: [None, 10, 50, 100]
RandomForestClassifier	“n_estimators”: [10, 50, 100], “max_depth”: [None, 10, 50, 100]

## Appendix 7: SWLS regression results for all feature sets

See Table 19.

**Table 19:** SWLS regression results for all feature sets

Features	Best model	Results		
		MAE	Pearson R	R-2
Mean baseline		0.1853	–	–
Median baseline		0.185	–	–
Words	ElasticNet	0.1744	0.3402	0.1022
RuLIWC	DecisionTree	0.182	0.2168	0.0142
AppCats	ElasticNet	0.1762	0.2737	0.0172
Behavior	DecisionTree	0.1785	0.191	0.0195
Clusters	RandomForest	0.1814	0.1709	0.026
AppCats + RuLIWC	ElasticNet	0.1776	0.2478	0.0296
AppCats + Behavior	ElasticNet	0.1784	0.2227	0.0248
AppCats + Words	Ridge	0.1756	0.2992	0.0864
RuLIWC + Behavior	DecisionTree	0.1818	0.1949	0.0133
RuLIWC + Words	ElasticNet	0.1722	0.352	0.0988
Behavior + Words	ElasticNet	0.1754	0.314	0.0752
clusters + AppCats	ElasticNet	0.1786	0.2545	0.0129
clusters + RuLIWC	DecisionTree	0.1769	0.2769	0.0507
clusters + Behavior	DecisionTree	0.1765	0.2243	0.0368
clusters + Words	Lasso	0.1715	0.3435	0.112
AppCats + RuLIWC + Behavior	ElasticNet	0.1761	0.3093	0.0704
AppCats + RuLIWC + Words	Lasso	0.1753	0.2913	0.0711
AppCats + Behavior + Words	ElasticNet	0.1735	0.3004	0.0724
RuLIWC + Behavior + Words	ElasticNet	0.1752	0.3506	0.0934
clusters + AppCats + RuLIWC	ElasticNet	0.1778	0.2636	0.0314
clusters + AppCats + Behavior	ElasticNet	0.1756	0.2341	0.0528
clusters + AppCats + Words	Lasso	0.1712	0.2958	0.0932
clusters + RuLIWC + Behavior	DecisionTree	0.1765	0.2275	0.038
clusters + RuLIWC + Words	ElasticNet	0.1712	0.3673	0.1192
clusters + Behavior + Words	ElasticNet	0.1712	0.3459	0.1228
clusters + AppCats + RuLIWC + Behavior	ElasticNet	0.1748	0.2962	0.0048
clusters + AppCats + RuLIWC + Words	Ridge	0.1751	0.2882	0.0811
*clusters* + *AppCats* + *Behavior* + *Words*	*ElasticNet*	**0.1698**	**0.4024**	**0.1045**
clusters + RuLIWC + Behavior + Words	Lasso	0.1776	0.294	0.0616
AppCats + RuLIWC + Behavior + Words	ElasticNet	0.1719	0.3255	0.096
*clusters* + *AppCats* + *RuLIWC* + *Behavior* + *Words*	*ElasticNet*	**0.1681**	**0.3776**	**0.1164**

## Appendix 8: WHO-5 regression results for all feature sets

See Table 20.

**Table 20:** WHO-5 regression results for all feature sets

Features	Best model	Results		
		MAE	Pearson R	R-2
Mean baseline		0.1542	–	–
Median baseline		0.1533	–	–
Words	Lasso	0.1441	0.3179	0.0817
RuLIWC	Lasso	0.1529	0.1276	0.0197
AppCats	ElasticNet	0.1511	0.2172	0.0329
Behavior	DecisionTree	0.1497	0.2463	0.0096
Clusters	Lasso	0.1516	0.1533	0.0241
AppCats + RuLIWC	Ridge	0.1505	0.2578	0.0371
AppCats + Behavior	Lasso	0.1458	0.2934	0.0678
AppCats + Words	ElasticNet	0.1458	0.3228	0.0772
RuLIWC + Behavior	DecisionTree	0.1505	0.2399	0.0032
RuLIWC + Words	Ridge	0.1445	0.3242	0.0964
Behavior + Words	AdaBoost	0.1473	0.2813	0.0476
clusters + AppCats	ElasticNet	0.1502	0.2537	0.0492
clusters + RuLIWC	AdaBoost	0.1527	0.1822	-0.007
clusters + Behavior	DecisionTree	0.15	0.2343	-0.0026
clusters + Words	ElasticNet	0.1449	0.2628	0.0975
clusters + AppCats + RuLIWC	ElasticNet	0.1493	0.2807	0.0786
clusters + AppCats + Behavior	ElasticNet	0.1469	0.3013	0.0739
clusters + AppCats + Words	Ridge	0.1444	0.338	0.0894
clusters + RuLIWC + Behavior	DecisionTree	0.1505	0.2399	0.0032
clusters + Behavior + Words	Ridge	0.1462	0.2389	0.0653
AppCats + RuLIWC + Behavior	ElasticNet	0.145	0.3363	0.0835
AppCats + RuLIWC + Words	Ridge	0.146	0.3222	0.0817
RuLIWC + Behavior + Words	ElasticNet	0.1479	0.2531	0.0531
AppCats + Behavior + Words	ElasticNet	0.1452	0.3152	0.0975
*clusters* + *RuLIWC* + *Words*	*AdaBoost*	**0.1436**	**0.3202**	**0.081**
clusters + AppCats + RuLIWC + Behavior	ElasticNet	0.1456	0.3394	0.0938
clusters + AppCats + RuLIWC + Words	ElasticNet	0.1472	0.3088	0.0716
clusters + AppCats + Behavior + Words	Lasso	0.1457	0.3339	0.0701
clusters + RuLIWC + Behavior + Words	ElasticNet	0.1478	0.2961	0.072
*AppCats* + *RuLIWC* + *Behavior* + *Words*	*ElasticNet*	**0.1438**	**0.367**	**0.1193**
clusters + AppCats + RuLIWC + Behavior + Words	ElasticNet	0.148	0.2952	0.0544

## Appendix 9: WHO-5 classification results

See Table 21.

**Table 21:** WHO-5 classification results

Classification	Threshold	N (Classes)	Features	Best model	F1-macro	F1-weighted	F1-low	F1-high	TruePositiveRate (low)	FalsePositiveRate (low)
binary	0.51	221/151	Words	AdaBoost	0.56	0.581	0.669	0.452	0.697	0.57
			RuLIWC	DecisionTree	0.571	0.582	0.631	0.512	0.611	0.457
			AppCats	AdaBoost	0.58	0.602	0.694	0.466	0.738	0.57
			Behavior	DecisionTree	0.543	0.559	0.63	0.456	0.638	0.55
			Clusters	RandomForest	0.539	0.571	0.714	0.363	0.832	0.715
binary majority baseline					0.378	0.456	0.373	0	1	1
trinary	0.35/0.59	111/158/103	Words	AdaBoost	0.44	0.447	0.407	0.43	0.378	0.195
			RuLIWC	AdaBoost	0.381	0.399	0.413	0.238	0.405	0.241
			AppCats	AdaBoost	0.422	0.443	0.402	0.294	0.396	0.241
			Behavior	AdaBoost	0.425	0.438	0.427	0.329	0.414	0.23
			Clusters	DecisionTree	0.358	0.364	0.338	0.339	0.351	0.295
			clusters + RuLIWC + Words	AdaBoost	0.483	0.493	0.502	0.433	0.45	0.161
trinary majority baseline					0.199	0.253	–	–	0	0

## Appendix 10: Features significant in SWLS regression

See Table 22.

**Table 22:** Features significant in SWLS regression

Feature	Mean importance	Count in 10-CV
спать_NOUN	41,086.4144049898	5
интим_NOUN	−44,937.4613019008	5
орг_NOUN	23,978.9614411828	5
дропнуть_VERB	−64,677.1586467715	5
тратиться_VERB	−24,593.5714641034	5
отл_UNKN	34,184.2112504721	5
пояснение_NOUN	−22,499.9757533852	5
стебать_VERB	−28,898.951393906	5
вифя_NOUN	−48,114.1470241285	5
спойлерить_VERB	−48,530.1211086886	5
ооохнуть_VERB	−44,864.4233831708	5
милый_COMP	56,128.262155605	5
пиздёжа_NOUN	−22,727.1849476408	5
Negative_month	−29.2652084171193	5
AppUsage9-12Ratio	10.3365760075427	5
SOCIAL+COMMUNICATION+DATING_0/SOCIAL+COMMUNICATION+DATING	11.9200141620517	5
AppUsage0-3Ratio	−8.02782185058373	5
обжечь_ VERB	−40,019.2136897226	5
PHOTOGRAPHY_6/6	8.00565760998601	5
объёмный_ADJ	−22,927.1436115299	4
разрывать_VERB	−30,217.4675429819	4
AppUsage6-9Ratio	6.14938364734453	4
Negative_year	−42.3845120787015	4
Negative_all	−31.9683341076574	4
(face-blowing-a-kiss_emoji)_UNKN	30.6632155496334	4
упад_NOUN	−18,580.4570156265	4
чонгук_ NOUN	−17,463.4313634737	4
дельфин_NOUN	21,536.5345583292	4
пиздуть_VERB	−14,962.8346296741	4
продумать_VERB	17,494.2319623544	4
PERSONALIZATION_3/3	7.00814836414381	4
385	24,539.3867498811	4
хл_UNKN	−16147.5539040561	4
TOOLS_2/2	6.32964889581622	4
блч_UNKN	−14,422.917489824	4
мразь_NOUN	−18,116.8461473664	4
ENTERTAINMENT_0/0	5.31480531809703	4
Percept_RuLIWC	−40.0983869406501	3
камбэк_ NOUN	−11,161.9164871315	3
помеха_ NOUN	16,006.6097736205	3
неудобный_ADJ	14,581.2712382097	3
байка_NOUN	−13,460.0791647949	3
но_CONJ	−33.8820301427059	3
бляяяяять_VERB	16,679.7792973482	3
OTHER_6/6	6.70178805009534	3
OTHER_6/OTHER	−5.92779708025781	3
OTHER_5/5	−6.64941501653413	3
OTHER_5/OTHER	7.9587777321838	3
пожениться_VERB	7984.73733958357	3
джуна_NOUN	−15,756.4913230161	3
хорошо_ ADV	27.4832618347005	3
расстроить_PRTF	10,055.3069444193	3
предать_VERB	9610.44789534047	3
критерий_NOUN	13,168.233814062	3
офф_UNKN	−16,763.6305231621	3
грубый_ ADJ	−9967.34916619578	3
съебывать_VERB	−14,161.996571157	3
фандом_ NOUN	−7058.65855912861	3
бляяяяяять_GRND	−8683.89310820064	3
PHOTOGRAPHY_4/4	−11.4270318169998	3
кореец_ NOUN	−8536.07198679033	3
бантан_ NOUN	11,240.4372555059	3
разреветься_VERB	9104.89644806333	3
GAME_0/GAME	3.63645809844946	3
EDUCATION+PRODUCTIVITY_1/1	−3.06771772638087	3
PERSONALIZATION_5/PERSONALIZATION	4.18099320731029	3
HEALTH+MEDICAL_7/HEALTH+MEDICAL	3.38255612173981	3
EDUCATION+PRODUCTIVITY_6/EDUCATION+PRODUCTIVITY	3.6103446132533	3
GAME_5/GAME	−3.60075795654176	3
SOCIAL+COMMUNICATION+DATING_1/SOCIAL+COMMUNICATION+DATING	6.3430323785416	3
HEALTH+MEDICAL_1/HEALTH+MEDICAL	3.65748802084215	3
GAME_4/GAME	6.1492563234405	3
PERSONALIZATION_6/6	−7.74392289361535	3
HEALTH+MEDICAL_4/4	−10.3948608039209	3
PERSONALIZATION_5/5	−4.53626915989602	3
PERSONALIZATION_6/PERSONALIZATION	5.56692243721861	3
EDUCATION+PRODUCTIVITY_3/EDUCATION+PRODUCTIVITY	−3.23127315840786	3
SOCIAL+COMMUNICATION+DATING_6/SOCIAL+COMMUNICATION+DATING	4.04158603764638	3
GAME_1/GAME	−7.22900142005906	3
PERSONALIZATION_3/PERSONALIZATION	−5.34430996987665	3
ENTERTAINMENT_1/ENTERTAINMENT	4.14113541946902	3
ENTERTAINMENT_2/2	4.31642268738505	3
PERSONALIZATION_0/PERSONALIZATION	−4.4149112724685	3
привет_ NOUN	23.4902884073796	2
EDUCATION+PRODUCTIVITY_4/EDUCATION+PRODUCTIVITY	0.992124886908841	2
Positive_month	25.7634204715238	2
EDUCATION+PRODUCTIVITY_5/EDUCATION+PRODUCTIVITY	1.05862884395041	2
TOOLS_5/TOOLS	−2.00490447501796	2
EDUCATION+PRODUCTIVITY_7/EDUCATION+PRODUCTIVITY	1.11603306580073	2
SOCIAL+COMMUNICATION+DATING_7/7	3.0908805432526	2
TOOLS_2/TOOLS	−6.49804253661374	2
TOOLS_4/TOOLS	−3.31388672845536	2
ENTERTAINMENT_2/ENTERTAINMENT	−2.54736564980811	2
SOCIAL+COMMUNICATION+DATING_7/SOCIAL+COMMUNICATION+DATING	−6.83408164115133	2
EDUCATION+PRODUCTIVITY_2/EDUCATION+PRODUCTIVITY	2.43891633479369	2
выпилиться_VERB	−3338.05050867729	2
EDUCATION+PRODUCTIVITY_2/2	2.59731699527922	2
еби_UNKN	19,201.5250764129	2
выглянуть_VERB	−7762.75345745476	2
гуглить_VERB	−1079.55071853441	2
растягивать_VERB	−5127.61602039587	2
жестокий_ADJ	−6724.2195734053	2
GAME_2/2	−2.50496667340079	2
заржать_VERB	−9032.15201413262	2
мэн_NOUN	18,667.8692410825	2
ENTERTAINMENT_4/4	−0.6931462276039	2
долбиться_VERB	−14,770.1618041756	2
петух_NOUN	−7131.85541414396	2
подробный_ADJ	6083.97042642484	2
оооохнуть_VERB	−12,538.581928229	2
загуглила_NOUN	−8903.85747472549	2
ущербный_ADJ	−10,188.6678026704	2
GAME_6/GAME	−0.557326373511503	2
EDUCATION+PRODUCTIVITY_0/EDUCATION+PRODUCTIVITY	1.32014414545248	2
See_RuLIWC	−44.9066092959057	2
TOOLS_1/TOOLS	0.246966591171979	2
SOCIAL+COMMUNICATION+DATING_0/0	0.145830646062444	2
HEALTH+MEDICAL_2/HEALTH+MEDICAL	−0.660450103454573	2
PHOTOGRAPHY_3/PHOTOGRAPHY	1.22028192527858	2
PHOTOGRAPHY_2/PHOTOGRAPHY	−3.17674625105626	2
PHOTOGRAPHY_7/PHOTOGRAPHY	−1.47432690185411	2
PHOTOGRAPHY_1/PHOTOGRAPHY	3.16110100642227	2
PHOTOGRAPHY_0/PHOTOGRAPHY	−2.35307771691311	2
OTHER_1/1	−1.69717416262808	2
OTHER_2/2	−1.36082437833807	2
OTHER_3/OTHER	4.37604917806843	2
SOCIAL+COMMUNICATION+DATING_4/4	−3.52025229911742	2
gender_merged	0.843233408502276	2
PHOTOGRAPHY_4/PHOTOGRAPHY	−1.33192379668633	2
HEALTH+MEDICAL_7/7	9.42472510509919	2
HEALTH+MEDICAL_6/HEALTH+MEDICAL	2.38143334031195	2
HEALTH+MEDICAL_4/HEALTH+MEDICAL	0.0719895107619945	2
SOCIAL+COMMUNICATION+DATING_6/6	3.03202297494442	2
HEALTH+MEDICAL_1/1	−12.9847665999778	2
GAME_0/0	−0.867851376244603	2
HEALTH+MEDICAL_0/HEALTH+MEDICAL	−1.41730497804968	2
PERSONALIZATION_4/PERSONALIZATION	−3.12105445284181	2
PERSONALIZATION_2/PERSONALIZATION	−1.24127259627768	2
PERSONALIZATION_7/PERSONALIZATION	1.51585802743145	2
TOOLS_4/4	−9.92596640160934	2
PERSONALIZATION_0/0	−3.19660012339845	2
ENTERTAINMENT_7/ENTERTAINMENT	−0.84084761762985	2
HEALTH+MEDICAL_5/HEALTH+MEDICAL	−0.129810361433915	1
шава_NOUN	−8941.24555908169	1
AppUsage15-18Ratio	−2.07642255991409	1
AppUsage21-24Ratio	−0.676863148867948	1
маркус_ NOUN	61,863.8448291371	1
ENTERTAINMENT_6/6	−13.5275610189105	1
научный_ADJ	4729.48292716799	1
ноооо_NOUN	5259.91641964248	1
намджуна_NOUN	−510.55327160631	1
AppUsage12-15Ratio	−10.37076321492	1
HEALTH+MEDICAL_3/3	26.6170643844024	1
ENTERTAINMENT_7/7	17.4976861479316	1
GAME_1/1	−0.0205129700590116	1
Alters_-9	−0.129041531649635	1
GAME_2/GAME	−2.71200941173272	1
EDUCATION+PRODUCTIVITY_4/4	0.177772668811767	1
TOOLS_0/0	−2.14285870492742	1
Alters_-7	0.153125018883583	1
TOOLS_6/TOOLS	2.69914996314137	1
OTHER_1/OTHER	−2.35449675924834	1
ENTERTAINMENT_3/3	1.1328494815993	1
PHOTOGRAPHY_1/1	0	1
ENTERTAINMENT_5/ENTERTAINMENT	0.149165800159887	1
ENTERTAINMENT_6/ENTERTAINMENT	−0.41391451812445	1
PERSONALIZATION_1/PERSONALIZATION	−0.330707466660351	1
HEALTH+MEDICAL_2/2	0.121502401677361	1
шов_NOUN	−4721.2176187302	1
бланк_NOUN	−4764.15988799968	1
GAME_4/4	−3.65092628597215	1
EDUCATION+PRODUCTIVITY_3/3	−6.80750290356738	1
ENTERTAINMENT_4/ENTERTAINMENT	−3.30081880604151	1
TOOLS_7/TOOLS	−4.04245464338458	1
TOOLS_5/5	−4.14302286116742	1
PERSONALIZATION_4/4	10.7567609647953	1
TOOLS_3/TOOLS	−4.12230296427837	1
TOOLS_0/TOOLS	−5.53385037327718	1
HEALTH+MEDICAL_3/HEALTH+MEDICAL	2.05274970971747	1
altersdiff	−0.844560300538125	1
HEALTH+MEDICAL_5/5	15.552182342051	1
EDUCATION+PRODUCTIVITY_0/0	−1.51789074810355	1
GAME_6/6	9.22791978620134	1
OTHER_7/OTHER	1.97270978786112	1
SOCIAL+COMMUNICATION+DATING_1/1	−0.103568092714721	1
потерянный_ADJ	−11,345.6959433921	1
саба_NOUN	2077.71458808275	1
SOCIAL+COMMUNICATION+DATING_5/5	−1.05176654037101	1
припереться_VERB	−5406.87753364421	1
OTHER_4/4	−5.5431060969613	1
OTHER_4/OTHER	3.93086530165725	1
OTHER_0/0	3.27602573972759	1
HEALTH+MEDICAL_6/6	15.2161412609318	1
писаться_VERB	−5296.54331114917	1
OTHER_0/OTHER	−2.08967236336632	1
поплакать_VERB	−318.617179611988	1
рэп_NOUN	−4852.08132918677	1
ложь_NOUN	6888.93905838351	1
PHOTOGRAPHY_5/PHOTOGRAPHY	0.837922272744407	1
growth-2to-1weighted	0.0682381400607952	1

## Appendix 11: Features significant in WHO-5 regression

See Table 23.

**Table 23:** Features significant in WHO-5 regression

Feature	Mean importance	Count in 10-CV
GAME_1/GAME	−5.30288559374647	7
ENTERTAINMENT_1/ENTERTAINMENT	4.48794365614162	7
HEALTH+MEDICAL_1/HEALTH+MEDICAL	2.6216421331719	6
AppUsage9-12Ratio	7.2634466399016	6
PERSONALIZATION_0/0	−3.93650446203669	6
EDUCATION+PRODUCTIVITY_3/EDUCATION+PRODUCTIVITY	−2.75547290725553	6
TOOLS_6/6	−3.38562106644281	5
SOCIAL+COMMUNICATION+DATING_1/SOCIAL+COMMUNICATION+DATING	7.08554306182447	5
GAME_3/GAME	2.11983623880978	5
OTHER_1/OTHER	−1.6572596556467	5
Bio_RuLIWC	−20.8118206754822	5
(face-blowing-a-kiss_emoji)_UNKN	35.1292524225535	5
EDUCATION+PRODUCTIVITY_7/EDUCATION+PRODUCTIVITY	−1.52932660473865	5
Negative_month	−32.9859591887424	5
Negative_year	−28.7441191861823	5
Negative_all	−22.8213190036261	5
но_CONJ	−16.0358199801479	5
ENTERTAINMENT_3/ENTERTAINMENT	1.89327664411053	5
PHOTOGRAPHY_0/PHOTOGRAPHY	−1.86907348608951	5
AppUsage6-9Ratio	3.86122248368424	4
See_RuLIWC	−17.771085104379	4
Percept_RuLIWC	−16.0075235125978	4
PHOTOGRAPHY_4/4	−11.8301205279096	4
SOCIAL+COMMUNICATION+DATING_7/SOCIAL+COMMUNICATION+DATING	5.36396284427798	4
OTHER_6/OTHER	−2.72825023219845	4
PERSONALIZATION_2/PERSONALIZATION	−2.65288208258266	4
хорошо_ ADV	11.8899128397086	4
HEALTH+MEDICAL_1/1	−12.7633565212712	4
PERSONALIZATION_0/PERSONALIZATION	−1.96354072113084	4
EDUCATION+PRODUCTIVITY_5/5	−8.71922494241247	4
gender_merged	1.72537700855949	4
PHOTOGRAPHY_4/PHOTOGRAPHY	−1.737331956631	4
EDUCATION+PRODUCTIVITY_4/EDUCATION+PRODUCTIVITY	1.25522498498395	4
ENTERTAINMENT_0/ENTERTAINMENT	−1.36074073571704	4
SOCIAL+COMMUNICATION+DATING_6/6	2.22405894249721	4
ENTERTAINMENT_6/ENTERTAINMENT	0.966349839431597	3
PHOTOGRAPHY_1/1	−4.02406844554479	3
OTHER_4/4	−2.7677868583523	3
OTHER_5/OTHER	3.85343503542729	3
PHOTOGRAPHY_1/PHOTOGRAPHY	3.71328277476559	3
PERSONALIZATION_1/PERSONALIZATION	−3.35252125337103	3
AppUsage15-18Ratio	−3.26882386884001	3
SOCIAL+COMMUNICATION+DATING_4/4	−3.31997843801445	3
HEALTH+MEDICAL_4/HEALTH+MEDICAL	−0.9428147681181	3
PERSONALIZATION_3/3	−3.84520578986224	3
HEALTH+MEDICAL_2/2	3.92987294169931	3
PHOTOGRAPHY_3/PHOTOGRAPHY	0.40923079097447	3
PHOTOGRAPHY_6/PHOTOGRAPHY	−1.29996830780878	3
OTHER_1/1	−0.784546722768581	3
altersdiff	−0.92538030647002	3
OTHER_0/OTHER	2.25557936917862	3
PHOTOGRAPHY_6/6	9.01538392975499	2
SOCIAL+COMMUNICATION+DATING_4/SOCIAL+COMMUNICATION+DATING	5.87503025553596	2
обжечь_ VERB	−48,599.526427015	2
офф_UNKN	−28,194.9413170442	2
PHOTOGRAPHY_0/0	3.92198345485137	2
SOCIAL+COMMUNICATION+DATING_5/SOCIAL+COMMUNICATION+DATING	−2.50586082868358	2
жестокий_ADJ	−21,716.6197777305	2
потерянный_ADJ	−20,316.8257664484	2
тратиться_VERB	−17,634.6227724749	2
PHOTOGRAPHY_7/PHOTOGRAPHY	−1.20678360573034	2
OTHER_0/0	−1.44570452070656	2
OTHER_3/OTHER	1.37984322453403	2
пригонять_VERB	21,225.458567208	2
дропнуть_VERB	−53,030.1050709822	2
OTHER_6/6	4.06504182009785	2
предать_VERB	36,199.9239128262	2
AppUsage12-15Ratio	−4.38636786177102	2
червь_NOUN	55,826.7353210136	2
ущербный_ADJ	−26,488.8457950415	2
ооохнуть_VERB	−20,570.0983686645	2
магнитный_ADJ	19,900.8586557732	2
оооохнуть_VERB	−34,380.0974521155	2
блч_UNKN	−19,552.7345641462	2
приобнять_VERB	49,086.8536049488	2
SOCIAL+COMMUNICATION+DATING_0/0	0.00300824222269163	2
вифя_NOUN	−22,048.0034645223	2
TOOLS_2/TOOLS	−1.55091403718346	2
growth-2to-1weighted	−0.506104865550831	2
вообще_ ADV	−5.14238891529362	2
привет_ NOUN	12.8226744206666	2
он_NPRO	−18.0820059246943	2
GAME_0/GAME	1.55931076688544	2
GAME_0/0	−1.05587959441984	2
GAME_3/3	−2.654246487825	2
PHOTOGRAPHY_5/5	17.2783798216844	2
HEALTH+MEDICAL_7/7	2.71003871145643	2
EDUCATION+PRODUCTIVITY_1/1	−1.52282930897709	2
EDUCATION+PRODUCTIVITY_2/EDUCATION+PRODUCTIVITY	−1.07500201275638	2
HEALTH+MEDICAL_0/0	3.6972143104661	2
Social_RuLIWC	21.8729634558609	2
TOOLS_3/TOOLS	0.807683500445982	2
TOOLS_6/TOOLS	1.50491110974576	2
ENTERTAINMENT_4/ENTERTAINMENT	−0.841292236238171	2
ENTERTAINMENT_7/ENTERTAINMENT	−0.53781828777184	2
PERSONALIZATION_1/1	2.05013057484557	2
PERSONALIZATION_2/2	2.41275356218853	2
PERSONALIZATION_4/PERSONALIZATION	−0.277615145486821	2
TOOLS_4/TOOLS	−0.332621565714857	2
PERSONALIZATION_6/PERSONALIZATION	1.98331184812083	2
ханна_NOUN	−20,365.7841289252	1
отбирать_VERB	−16,422.664060777	1
шлюшка_ NOUN	7462.2668479906	1
интим_NOUN	−8415.75871614007	1
отл_UNKN	18,240.2790750433	1
бабочка_NOUN	22,242.8428202378	1
кпоп_NOUN	−22,706.902332252	1
объёмный_ADJ	−30,296.798373289	1
упад_NOUN	−17,378.1196878735	1
анимешник_NOUN	−8288.74761112379	1
хотя_CONJ	−2.32750500704937	1
критерий_NOUN	37,329.1994647902	1
слишком_ADV	−2.67847601625444	1
AppUsage18-21Ratio	3.02994634652915	1
EDUCATION+PRODUCTIVITY_7/7	3.4977040679328	1
выглянуть_VERB	−19,143.9898454013	1
хдд_UNKN	−2.41764183408381	1
PERSONALIZATION_3/PERSONALIZATION	−1.45062334734687	1
загуглила_NOUN	38,940.8898266739	1
HEALTH+MEDICAL_2/HEALTH+MEDICAL	−1.70766506251123	1
SOCIAL+COMMUNICATION+DATING_1/1	0.214043849547984	1
SOCIAL+COMMUNICATION+DATING_7/7	0.118134328089427	1
GAME_6/GAME	0.334957523880728	1
GAME_7/7	2.06442883253146	1
EDUCATION+PRODUCTIVITY_3/3	1.17072928106647	1
TOOLS_0/0	0.690890866736	1
TOOLS_5/5	1.77062991115663	1
TOOLS_7/TOOLS	1.2692618820954	1
ENTERTAINMENT_3/3	4.91803252713477	1
PERSONALIZATION_5/PERSONALIZATION	−0.015250988462515	1
HEALTH+MEDICAL_5/HEALTH+MEDICAL	−0.949406780362511	1
Alters_-7	0.0266628614988393	1
SOCIAL+COMMUNICATION+DATING_2/SOCIAL+COMMUNICATION+DATING	−1.16690389645055	1
SOCIAL+COMMUNICATION+DATING_6/SOCIAL+COMMUNICATION+DATING	−3.05422385174122	1
PHOTOGRAPHY_2/2	0.335187657879573	1
PHOTOGRAPHY_5/PHOTOGRAPHY	1.93680325337435	1
OTHER_4/OTHER	2.1244521858398	1
OTHER_5/5	−2.29647607260118	1
OTHER_7/7	2.51738861629993	1
AppUsage0-3Ratio	−2.10969417137118	1
GAME_1/1	−1.06574716160273	1
PERSONALIZATION_7/7	0	1
джуна_NOUN	−45,463.0625569559	1

## Footnotes

1 https://github.com/dlatk/happierfuntokenizing.

2 https://arxiv.org/abs/1804.10742, the code https://github.com/Kipok/clr_prediction was modified and applied.

3 https://naeglelab.github.io/OpenEnsembles/_modules/finishing.html#majority_vote

## Abbreviations

AUC: Area Under the Curve
DF: DigitalFreud
DSM-5: Diagnostic and Statistical Manual of mental disorders, fifth edition
GAD: General Anxiety Disorder scale
PSS: Perceived Stress Scale
LIWC: Linguistic Inquiry & Word Count dictionary
MAE: Mean Absolute Error
NLP: Natural Language Processing
PHQ-9: Patient Health Questionnaire
RFE: Recursive Feature Elimination
RMSE: Root-Mean-Square Error
RuLIWC: Russian Linguistic Inquiry & Word Count
RuSentiLex: Russian Sentiment Lexicon
SWB: Subjective Well-Being;
SWLS: Diener’s Satisfaction with Life Scale
TFIDF: Term Frequency Inverse Document Frequency
WHO-5: World Health Organization-5 Well-Being Index
